# Atypical memory B cells acquire Breg phenotypes in hepatocellular carcinoma

**DOI:** 10.1172/jci.insight.187025

**Published:** 2025-02-25

**Authors:** Shi Yong Neo, Timothy Wai Ho Shuen, Shruti Khare, Joni Chong, Maichan Lau, Niranjan Shirgaonkar, Levene Chua, Junzhe Zhao, Keene Lee, Charmaine Tan, Rebecca Ba, Janice Lim, Joelle Chua, Hui Shi Cheong, Hui Min Chai, Chung Yip Chan, Alexander Yaw Fui Chung, Peng Chung Cheow, Prema Raj Jeyaraj, Jin Yao Teo, Ye Xin Koh, Aik Yong Chok, Pierce Kah Hoe Chow, Brian Goh, Wei Keat Wan, Wei Qiang Leow, Tracy Jie Zhen Loh, Po Yin Tang, Jayanthi Karunanithi, Nye Thane Ngo, Tony Kiat Hon Lim, Shengli Xu, Ramanuj Dasgupta, Han Chong Toh, Kong-Peng Lam

**Affiliations:** 1 Singapore Immunology Network (SIgN), Agency for Science, Technology and Research (A*STAR), Singapore.; 2 Department of Oncology and Pathology, Karolinska Institute, Stockholm, Sweden.; 3 Division of Medical Oncology, National Cancer Centre Singapore, Singapore.; 4 Laboratory of Precision Oncology and Cancer Evolution, Genome Institute of Singapore (GIS), Agency for Science, Technology and Research (A*STAR), Singapore.; 5 Department of Hepato-pancreato-biliary and Transplant Surgery, Singapore General Hospital, Singapore.; 6 Division of Surgery and Surgical Oncology, National Cancer Centre Singapore, Singapore.; 7 Department of Anatomical Pathology, Singapore General Hospital, Singapore.; 8 Department of Physiology, Yong Loo Lin School of Medicine, National University of Singapore, Singapore.; 9 School of Biological Sciences, Nanyang Technological University, Singapore.; 10 Department of Microbiology & Immunology, Yong Loo Lin School of Medicine, National University of Singapore, Singapore.

**Keywords:** Hepatology, Immunology, Adaptive immunity

## Abstract

The functional plasticity of tumor-infiltrating lymphocyte B–cells (TIL-B) spans from antitumor responses to noncanonical immune suppression. Yet, how the tumor microenvironment (TME) influences TIL-B development is still underappreciated. Our current study integrated single-cell transcriptomics and B cell receptor (BCR) sequencing to profile TIL-B phenotypes and clonalities in hepatocellular carcinoma (HCC). Using trajectory and gene regulatory network analysis, we were able to characterize plasma cells and memory and naive B cells within the HCC TME and further revealed a downregulation of BCR signaling genes in plasma cells and a subset of inflammatory TNF^+^ memory B cells. Within the TME, a nonswitched memory B cell subset acquired an age-associated B cell phenotype (TBET^+^CD11c^+^) and expressed higher levels of PD-L1, CD25, and granzyme B. We further demonstrated that the presence of HCC tumor cells could confer suppressive functions on peripheral blood B cells that in turn, dampen T cell costimulation. To the best of our knowledge, these findings represent novel mechanisms of noncanonical immune suppression in HCC. While previous studies identified atypical memory B cells in chronic hepatitis and across several solid cancer types, we further highlighted their potential role as regulatory B cells (Bregs) within both the TME and peripheral blood of HCC patients.

## Abstract 2

Atypical memory B cells can be immunosuppressive and found within peripheral blood and the tumor microenvironment of hepatocellular carcinoma patients.

## Graphical abstract

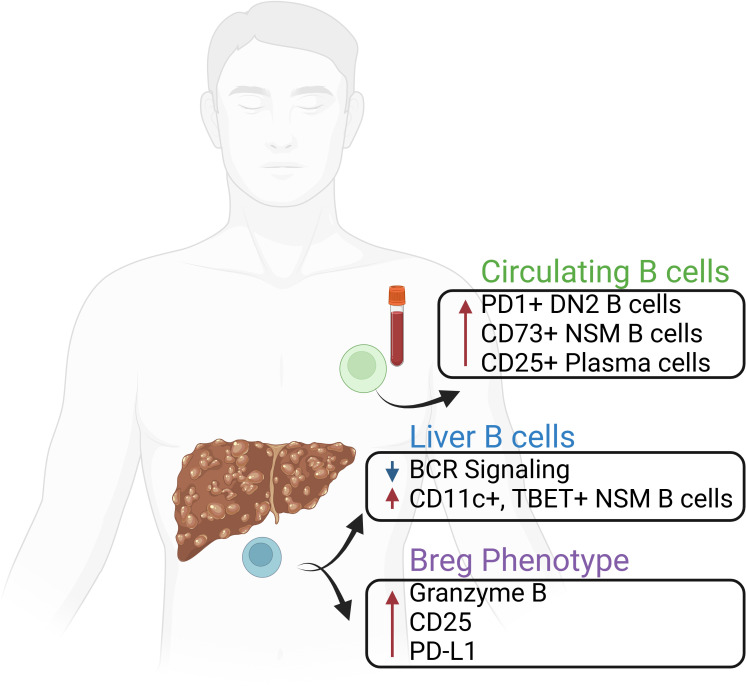

## Introduction

Across cancers, tumor-infiltrating lymphocyte B–cells (TIL-B) and plasma cells contribute to antitumor immunity and could serve as a prognostic marker, particularly in virus-driven cancers such as those associated with human papillomavirus (HPV), hepatocellular carcinoma (HCC), and Epstein-Barr virus (EBV)-driven squamous cell carcinomas (1–3). For instance, the density of TIL-B and T cell interactions could serve as a functional biomarker for improved survival of patients with HCC (1). On the other hand, TIL-B cells can also be associated with poor prognosis attributable to their regulatory B cell (Breg) role of immune tolerance. An early study reported that circulating Bregs and intrahepatic B cells correlated with advanced tumor stage and tumor recurrence in HCC, respectively (4). Other subsequent studies of HCC revealed that PD-1^hi^, IL-10–producing Bregs were similarly associated with early disease recurrence, while PD-L1^+^ plasma cells secrete IgA to promote fibrosis, potentially aggravating metabolic dysfunction–associated steatohepatitis–HCC (MASH-HCC) (5, 6). Although the activation of B cells by hepatitis B virus (HBV) antigen has been well characterized (7–9), how HCC differs from chronic hepatitis in influencing the phenotype and function of TIL-B cells remains unclear.

Increasing evidence indicates that Bregs are profound suppressors of NK- and T cell–mediated antitumor responses and could be activators of regulatory T cells (Tregs) and myeloid-derived suppressor cells (MDSCs) (10–12). But unlike Tregs, there remains no consensus on the developmental trajectory of Bregs, although they are known to express IL-10, IL-35, and TGF-β within the TME (13, 14). Our present study sought to profile B cell phenotypes and identified Breg cell subsets within peripheral blood and tissues of patients with HCC. We further demonstrated that peripheral blood B cells could be influenced by HCC tumor cells to upregulate TBET and acquire regulatory functions that dampen T cell costimulation.

## Results

### Examination of B cell phenotypes in virus-associated HCC through the integration of single-cell transcriptomics and BCR profiling.

**Figure 1:**
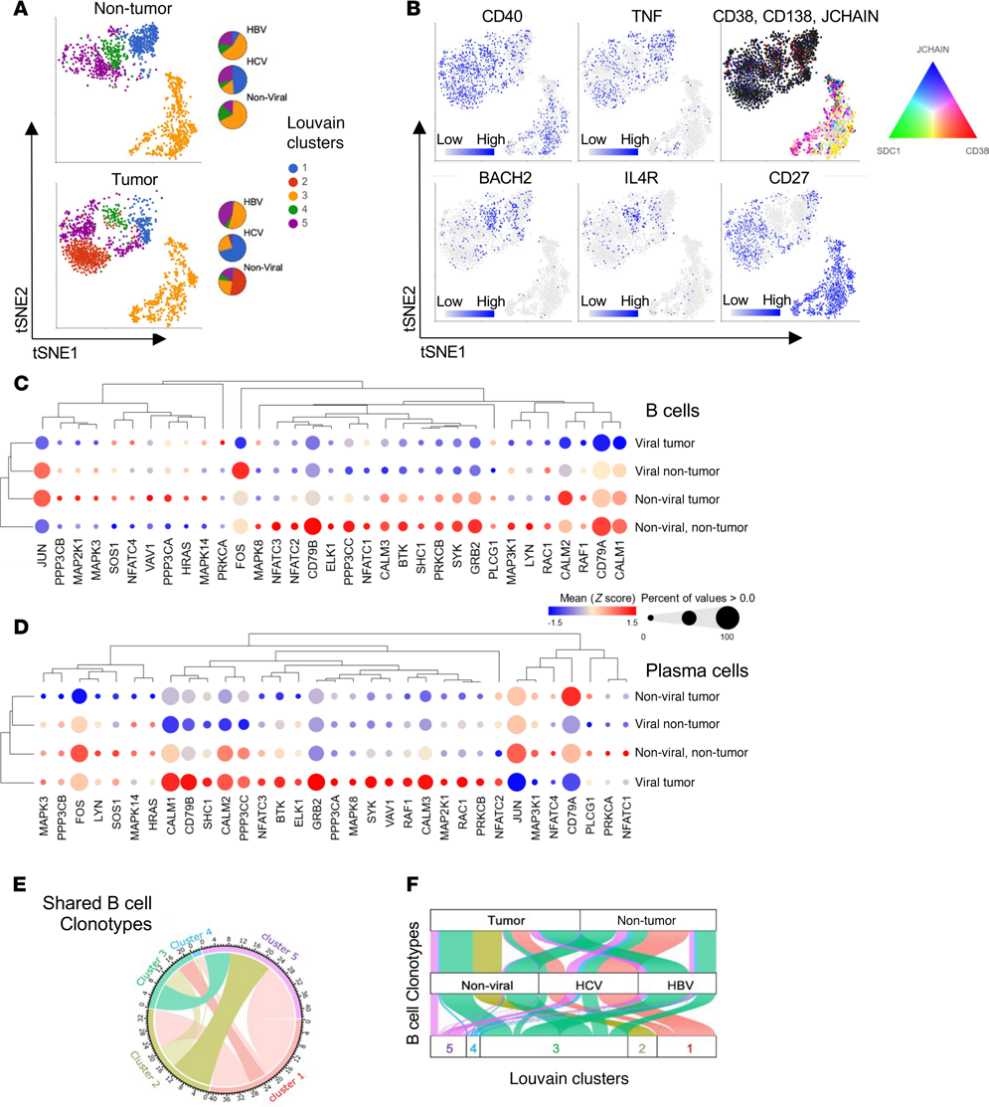
Examination of B cell phenotypes in HCC through integration of single-cell transcriptomics and BCR profiling. (**A**) tSNE projection of B cell subsets (clusters 1, 2, 4, and 5) and plasma cells (cluster 3) based on Louvain clustering and pie charts showing their distributions within nontumor and tumor sample types of different viral status. (**B**) tSNE projections of B cell subsets and plasma cells with their differentially expressed gene (DEG) features. Bubble heatmaps with unsupervised hierarchical clustering showing normalized expression of BCR-related genes (Biocarta, M9494) expressed by (**C**) B cells and (**D**) plasma cells isolated from various types of HCC resected tissues. (**E**) Chord diagram showing shared B cell clonotypes among the 5 clusters. (**F**) Alluvial plot illustrating the distribution of BCR clonotypes with respect to tumor and viral status, as well as denoting clusters identified from the gene expression data. In **E** and **F**, the proportions of BCR clonotypes within each B cell phenotype are represented by the width of the corresponding colored bands.

We first characterized B and plasma cells by single-cell transcriptomics from tumor and nontumor tissues obtained from surgical resections in a cohort of 10 patients with HCC (patient characteristics described in Supplemental Table 1; supplemental material available online with this article; https://doi.org/10.1172/jci.insight.187025DS1). Using Louvain clustering, we identified a cluster of plasma cells (cluster 3) and 4 clusters of B cells with distinct differentially expressed genes (DEGs) distributed in both nontumor and tumor tissues of different viral status (Figure 1, A and B, and Supplemental Figure 1A). While clusters 1, 3, 4, and 5 were B cells found in nontumor and tumor samples (Supplemental Figure 1B), cluster 2 was notably found only in 1 nonviral HCC tumor (8T), which was identified to be a memory B cell cluster that coexpresses *IRF8* and *CD82*. Cells in cluster 2 also highly express *FABP1* and are enriched for genes related to cholesterol and triglyceride metabolism (Supplemental Figure 1, C and D). Comparing viral versus nonviral HCC tissues, B cells in general downregulated multiple genes encoding proteins involved in the B cell receptor (BCR) signaling pathway (Figure 1C and Supplemental Figure 2, A and B). Of note, several of these BCR-related genes were also differentially expressed comparing tumor B cells to nontumor B cells within the nonviral group (Figure 1C). Components of the AP-1 subunits (*JUN, JUNB, FOS*, *FOSB*) were also downregulated in B cells of HBV-HCC and HCV-HCC tumors but the opposite trend was observed in B cells residing in nontumor tissues (Supplemental Figure 2, A and B). Interestingly, a large proportion of BCR-related genes were upregulated in plasma cells from viral tumors and particularly in HBV-HCC (Figure 1D and Supplemental Figure 2, A and B). In contrast, plasma cells within nontumor tissue revealed an opposite trend whereby the downregulation of BCR-related genes was more prominent only in HBV-HCC tissues (Supplemental Figure 2A). Next, we integrated BCR sequencing into our transcriptomics dataset to reveal a network of B cell clonotypes that were shared extensively among the various clusters except for cluster 4 (Figure 1E). Within the 5 B cell clusters, traceable BCR clonotypes could be identified in both viral and nonviral tissues, while clonotypes in cluster 2 were limited to nonviral tumors (Figure 1F).

To substantiate our findings, we examined another publicly available dataset of HCC single-cell transcriptomics (NCBI GEO GSE149614) to confirm whether similar downregulation of BCR-related genes was observed (15). Despite having 11 subclusters of B cells identified, the majority of the B cells in this study were isolated from nonviral samples and moreover, B cells from HCV-HCC samples were obtained from nontumor tissues (Supplemental Figure 3, A–C). Focusing on only B cells in nontumor tissues, we similarly observed downregulation of BCR signaling–related genes in HBV-HCC and HCV-HCC as compared with nonviral HCC (Supplemental Figure 3, D and E) samples. Collectively, we profiled the differential landscape of B cell phenotypes and clonalities, uncovering a downregulation of BCR signaling genes in virus-associated HCC.

### Trajectory and gene regulatory network analyses revealed differential transcriptional programs within B cell subsets in HCC.

**Figure 2:**
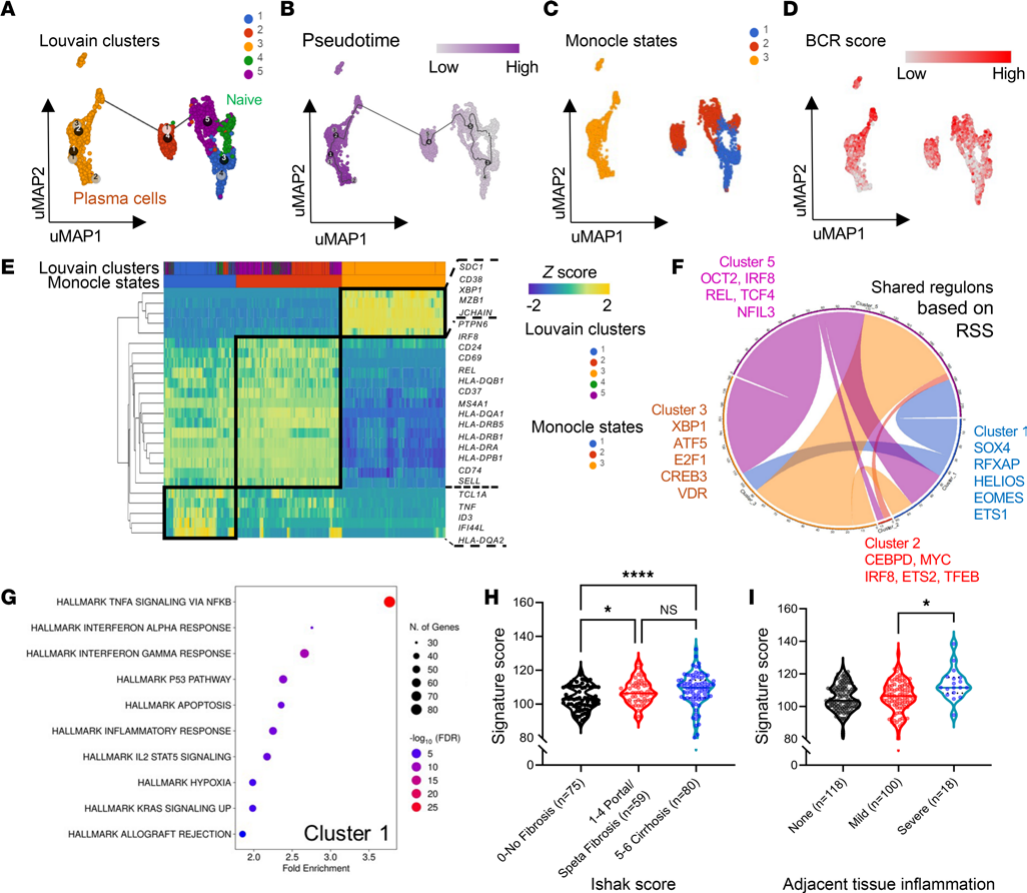
Trajectory and gene regulatory network analyses revealed differential transcriptional programs within B cell subsets found in HCC. UMAP projections of monocle trajectory analysis showing the (**A**) Louvain clusters, (**B**) pseudotime analysis, (**C**) monocle states, and (**D**) relative expression score for BCR signaling pathway (Biocarta, M9494). (**E**) Hierarchical heatmap clustering of significant DEGs based on monocle state-of-trajectory analysis. (**F**) Chord diagram of common regulons between clusters based on the SCENIC method. Thickness of the connections between clusters depends on number of shared regulons between them. The top 5 relevant regulons with a regulon specificity score (RSS) cutoff of 0.35 were used for each Louvain cluster. (**G**) Gene enrichment was done based on top 200 differentially expressed genes with reference to mSigDB Hallmark gene sets. Only the top 10 significant gene sets are shown. Expression of normalized gene signature score in bulk primary tumors of the TCGA liver cancer cohort (LIHC) classified based on Ishak fibrosis score (**H**) and adjacent tissue inflammation scoring (**I**). Kruskal-Wallis test was used to determine significance. *P < 0.05; ****P < 0.0001. NS, not significant.

To further dissect the relationship of various B cell subsets, we first identified cluster 4 to resemble naive-like B cells (IL-4R^+^BACH2^+^CD27^–^) with the least detected BCR clonotypes, unlike other B cell subsets (Figure 1, B and E). We next projected the various B cell clusters in a pseudotime trajectory analysis, clustering plasma cells and memory and naive B cells into 3 main monocle states (Figure 2, A–C). Notably, we uncovered that a subset of B cells (monocle state 1, Louvain cluster 1) and a portion of plasma cells (monocle state 3) have a low expression score of the BCR signaling gene set (Figure 2D). Likewise, genes such as *SELL* (CD62L), *SHP1* (PTPN6), *IRF8*, and *CD24* were also downregulated in monocle state 1, as observed within the relevant trajectory–derived DEGs (Figure 2E). Using the single-cell regulatory network inference and clustering (SCENIC) algorithm (16), we next investigated the gene regulatory networks within the 5 B and plasma cell clusters, identifying the top predicted regulons in which some are shared among plasma and memory B clusters (clusters1, 2, 3, and 5) but not in cells of cluster 4 (Figure 2F and Supplemental Figure 4).

While it was previously reported that plasma cells contributed to the development of MASH-HCC (6), the role of memory B cells within the TME remains largely unknown. Here, we continue to focus on B cells with a low BCR signaling score (monocle state 1, Louvain cluster 1) and found that they expressed *TNF* (Figure 1B and Figure 2E). Similar to plasma cells, these TNF^+^ B cells upregulated genes encoding the AP-1 transcriptional complex, with a marked downregulation of *CD79* and other genes involved in BCR signaling (Supplemental Figure 5A). Additionally, B cells in cluster 1 were also enriched for inflammatory gene sets that were not observed in other clusters (Figure 2G and Supplemental Figure 5, B–E). Using a publicly available dataset of patients receiving anti–PD-1 therapy, we identified a B cell subset (cluster 2) associated with tolerance to immunotherapy (17) that was similarly enriched for inflammatory gene sets like HCC B cell cluster 1 defined here (Supplemental Figure 6, A and B). With reference to baseline samples, intratumoral B cells upregulated *JUN* and *FOS* only in patients with favorable responses to therapy, while B cells in nonresponders expressed higher levels of multiple BCR-related genes, including *SYK*, *CD79A*, and *CD79B* (Supplemental Figure 6C). Comparing these inflammatory B subsets from both cohorts, common DEGs could be identified in which we notably found 16 genes to be highly expressed (Supplemental Figure 6, D and E). Applying this set of DEGs (except *FCMR* and *CEMIP2*) as a Breg signature for immune deconvolution, we next examined the TCGA liver cancer cohort for correlation analysis into clinico-histopathological features. HCC tumors with higher signature scores were associated with increased fibrosis (Figure 2H) and inflammation in adjacent normal tissues (Figure 2I). Further GSEA revealed HCC tumors with higher signature scores were genetically enriched for inflammatory and EMT pathways (Supplemental Figure 6F). Collectively, our analysis here further uncovered the development trajectory and gene signature of an inflammatory B cell subset that is associated with distinct transcriptional and pathological tumor features.

### Patient-derived HCC organoids modulate B cell functionality in vitro.

**Figure 3:**
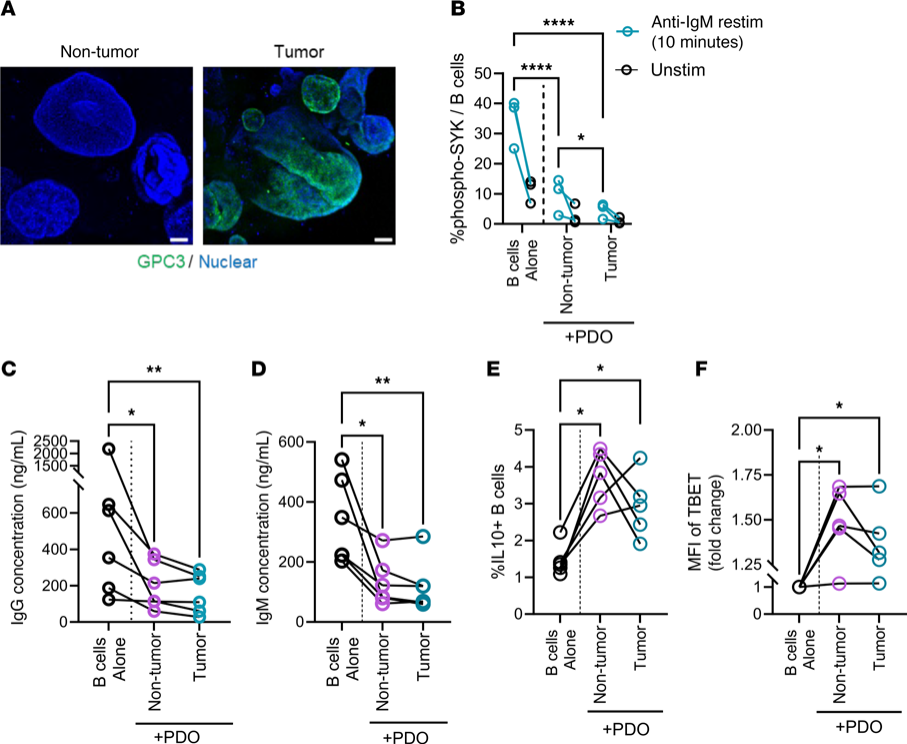
Patient-derived HCC organoids modulate B cell functionality in vitro. (**A**) Representative immunofluorescence of patient-derived tumor and nontumor organoids stained for the expression of GPC3 (tumor marker) under ×10 objective magnification. Scale bars: 80 μm. (**B**) Percentage of B cells positive for p-SYK as measured by flow cytometry after 3 days of allogenic cocultures with patient-derived organoids (PDOs). Anti-IgM was used to restimulate B cells for 10 minutes after 3 days of PDO–B cell coculture. 24. In **B**, 2-way ANOVA with multiple comparison using Fisher’s LSD test was used to test for significance (*n* = 3 biological replicates). Concentration of (**C**) IgG and (**D**) IgM in supernatants collected from 3-day cocultures of PDOs and B cells. In **C** and **D**, Friedman’s test with multiple comparison was used (*n* = 6 biological replicates). (**E**) Frequencies of IL-10–producing B cells after overnight treatment with brefeldin A and monensin after 3 days of PDO coculture. (**F**) Relative mean fluorescence intensity (MFI) of TBET expression after 3 days of PDO coculture normalized to fold change of untreated B cells cultured alone. In **C**–**F**, Friedman’s test with multiple comparison for Dunn’s test was used. In **E** and **F**, repeated-measures 1-way ANOVA with multiple comparisons was used to test for significance (*n* = 5 biological replicates). *P < 0.05; **P < 0.01; ****P < 0.0001.

While the differential gene expression analysis provided a general profile for B cells in the TME, we sought to better understand their function through the use of in vitro functional assays. We first generated patient-derived organoids (PDOs) from tumor and nontumor tissues of nonviral HCC origin (Figure 3A). The presence of either nontumor or tumor PDOs dampened the phosphorylation of SYK in response to anti-IgM stimulation. Corroborating our transcriptome findings (Figure 1C), there was nonetheless a significantly higher proportion of p-SYK^+^ B cells in nontumor PDO than in tumor PDO cultures (Figure 3B). As determined from the supernatants of these PDO–B cell cocultures, the production of IgG and IgM was downregulated in the presence of either tumor or nontumor PDOs as compared with the control (no PDO) group (Figure 3, C and D). While the presence of PDOs influenced BCR stimulation and antibody production, we also noticed that both IL-10 and TBET expression was upregulated by B cells in the presence of either nontumor or tumor PDOs (Figure 3, E and F). Altogether, it should be highlighted that nontumor cells from HCC patients should not be regarded as “normal” and our in vitro system here further demonstrated how B cells can be functionally modulated by both nontumor and tumor HCC organoids in a similar manner.

### Distinct atypical memory and Breg phenotypes could be found within the HCC microenvironment.

**Figure 4:**
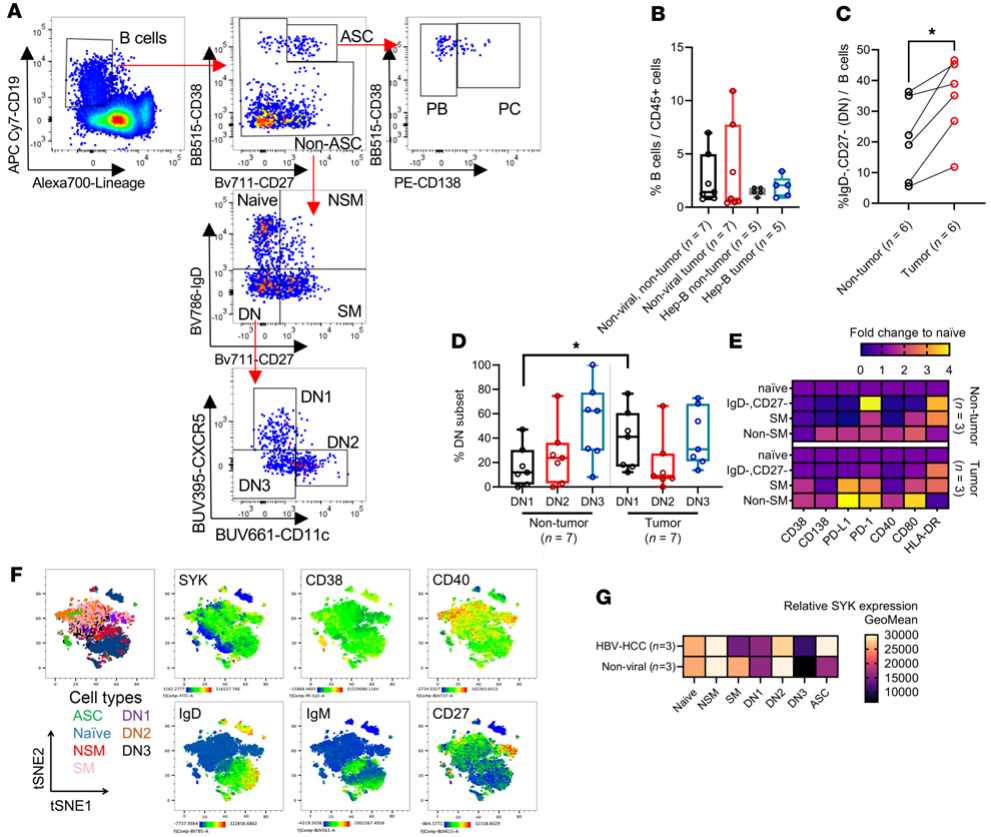
Distinct atypical memory B cell and Breg phenotypes could be found within the HCC microenvironment. (**A**) Flow cytometric dot plots for the gating strategy of various B and plasma cell subsets. ASC, antibody-secreting cells; PC, plasma cells; PB, plasmablasts; Non-SM, nonswitched memory; SM, switched memory; DN, double negative. (**B**) Percentage of CD19^+^ B cells within the immune compartment of various HCC tissues. (**C**) Frequencies of IgD^–^CD27^–^ DN cells versus total B cells comparing nontumor and tumor. (**D**) Proportions of DN1 (CXCR5^+^CD11c^–^), DN2 (CXCR5^–^CD11c^+^), and DN3 (CXCR5^–^CD11c^–^) within the DN B cell populations. In **C** and **D**, Wilcoxon’s signed-rank test was used to test for significance in the comparison of nontumor to tumor tissues. *P < 0.05. (**E**) Heatmap showing expression of various phenotypic markers in various B cell subsets normalized to naive B cells. (**F**) tSNE projections of various B cell subsets from flow cytometric phenotyping (*n* = 6 tumors). (**G**) Relative expression of SYK in various B cell subsets as measured by geometric mean of fluorescence intensity. Sample sizes reported in labels of all graphs and heatmaps.

We next collected a second cohort of HCC samples (described in Supplemental Table 2) to perform much more definitive classification of plasma cells and various naive and memory B subsets by flow cytometry. Due to the scarcity of HCV-HCC samples attributed to our local demographics (18), we focused on nonviral and HBV-HCC samples for further B cell profiling. Nonetheless, we were able to identify various plasma and B cell subsets (Figure 4A). Despite no differences in the frequencies of total B cells within tumor and nontumor tissues of nonviral HCC and HBV-HCC patients, we noticed a greater variability of B cell frequencies within tissues of nonviral patients (Figure 4B). Previous studies in autoimmunity revealed a subset of atypical memory B cells to be CD27^–^ and IgD^–^ double negative (DN) that is commonly associated with inflammatory and regulatory functions (19, 20). Here, we observed that the proportion of the DN B cell subset was significantly increased in tumor as compared with nontumor tissues (Figure 4C). In particular, the DN1 B subset was increased in tumors while the proportions of DN2 and DN3 were similar in nontumor and tumor tissues (Figure 4D). Comparison of nontumor to tumor B cells revealed that the DN B cells highly expressed PD-1 only within nontumor tissues and to our surprise, nonswitched memory (NSM) and switched memory (SM) B cells highly expressed both PD-L1 and PD-1 in tumor compared with nontumor tissues (Figure 4E). In a separate experiment, we also sought to confirm our transcriptomics data (Figure 1C) and observed similar downregulation of SYK in SM B cells in HBV-HCC tumors as compared with nonviral tumors (Figure 4, F and G). Also corroborating the transcriptomics data on plasma cells (Figure 1D), increased SYK expression in HBV-HCC plasma cells was observed (Figure 4G). While the atypical memory DN cells were found to be dysfunctional in chronic hepatitis and across various cancers (21–23), we identified and speculated here that perhaps PD-L1^+^ NSM and SM B cells could have a more prominent Breg phenotype within the TME of HCC.

### HCC-associated Breg phenotypes are inducible upon exposure to tumor cells.

**Figure 5:**
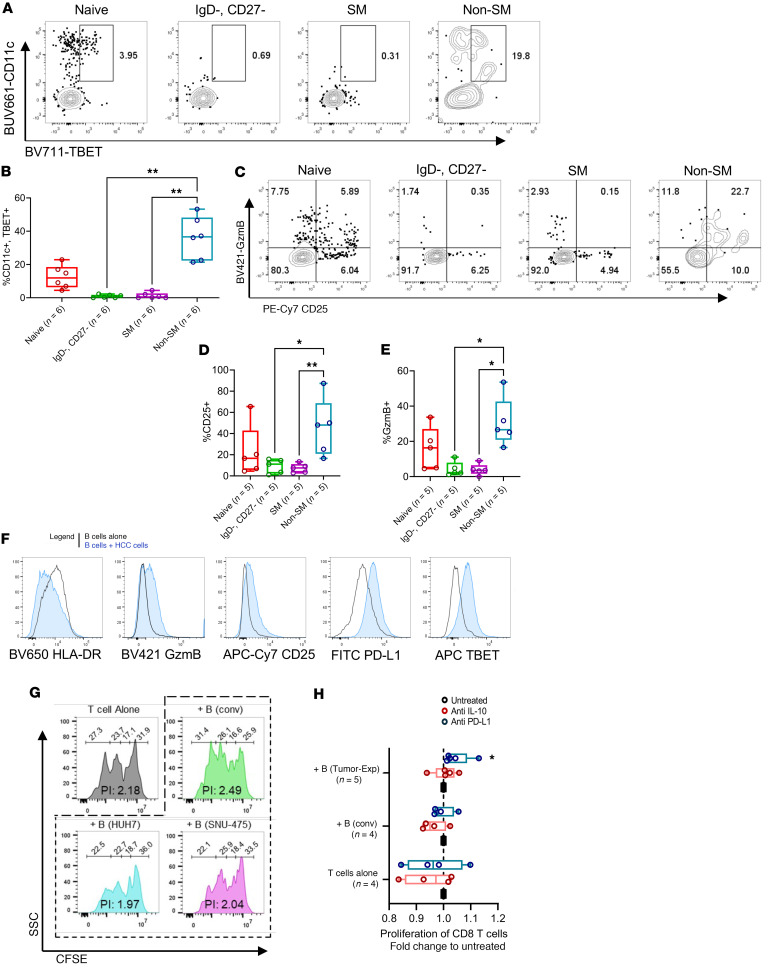
HCC-associated Breg phenotypes are inducible upon exposure to tumor cells. (**A**) Representative FACS plots for the expression of TBET and CD11c in various B cell subsets. (**B**) Frequencies of TBET^+^ and CD11c^+^ double-positive cells within various subsets of B cells. (**C**) Representative FACS plots for the expression of granzyme B (GzmB) and CD25 in various subsets of B cells. Frequencies of (**D**) CD25^+^ and (**E**) GzmB^+^ cells within various subsets of B cells. (**F**) Representative flow cytometry histograms (biological replicates, *n* < 4) for the expression of HLA-DR, GzmB, CD25, TBET, and PD-L1 after 3 days of coculturing B cells with the HCC tumor cell line HUH7 or SNU475 (also refer to Supplemental Figure 6, A–E). (**G**) Representative flow cytometry histograms (biological replicates, *n* = 5) for CFSE-labelled CD8^+^ T cells for calculation of proliferation index (also refer to Supplemental Figure 6F). (**H**) Proliferation of CD8^+^ T cells cotreated with either an IL-10–neutralizing antibody or anti–PD-L1 (atezolizumab) normalized to untreated control (see Methods for details of inhibitor treatment). Proliferation index was normalized to T cell–alone control. Kruskal-Wallis test was used to test for significance (**B**, **D**, **E**, and **H**). *P < 0.05; **P < 0.01. Sample sizes reported in labels of all graphs and heatmaps.

In recent years, inflammatory B cells have garnered much interest for their identity as age-associated B cells (ABCs) and are commonly recognized to be CD11c^+^ and TBET^+^ double positive in autoimmunity (24). While a recent study detected similar ABCs as atypical memory B cells within the TME (21), there remains a lack of understanding of ABCs and their specific roles particularly in human immunobiology. We found here that a larger proportion of CD11c^+^TBET^+^ cells were the NSM B cells within HCC tissues (Figure 5, A and B). At the same time, NSM B cells also highly expressed CD25 and granzyme B (Figure 5, C–E). While the expression of granzyme B in B cells was known to suppress T cells in tumors (25), the role of CD25 in Bregs here remains elusive but could plausibly act as a local sink for IL-2, similar to Treg-mediated immune suppression. Activated naive B cells were previously known to acquire similar ABC-associated markers (26). Notably, we also observed a trend where a proportion of naive-like B cells were also CD11c^+^TBET^+^ double positive (Figure 5B) and expressed CD25 and granzyme B (Figure 5, D and E).

Several studies demonstrated how suppressive immune cell types such as MDSCs can be induced by coculture with tumor cells (27). As such, we examined whether a similar approach could also generate Bregs ex vivo that acquire suppressive functions. Similar to a previous study on suppressive monocytes (27), we found B cells to also downregulate HLA-DR upon HCC coculture and as hypothesized, the presence of HCC tumor cells led to increased production of granzyme B in B cells, together with an upregulation of CD25, TBET, and PD-L1 (Figure 5F and Supplemental Figure 7, A–E). Furthermore, the ability to costimulate CD^+^8 T cells was reduced in tumor-experienced B cells compared with conventional B cells (Figure 5G and Supplemental Figure 7H) and this attenuation of B cell function could be partially rescued with PD-L1 inhibition rather than IL-10 neutralization (Figure 5H). Although not significant, similar trends were observed for the proliferation of CD4^+^ T cells (Supplemental Figure 7I). Collectively, we demonstrated here that tumor-experienced B cells in vitro could acquire a Breg phenotype as observed in NSM B cells profiled from HCC tissues.

### Expansion of DN2 and NSM B cells in peripheral blood of virus-driven HCC.

**Figure 6:**
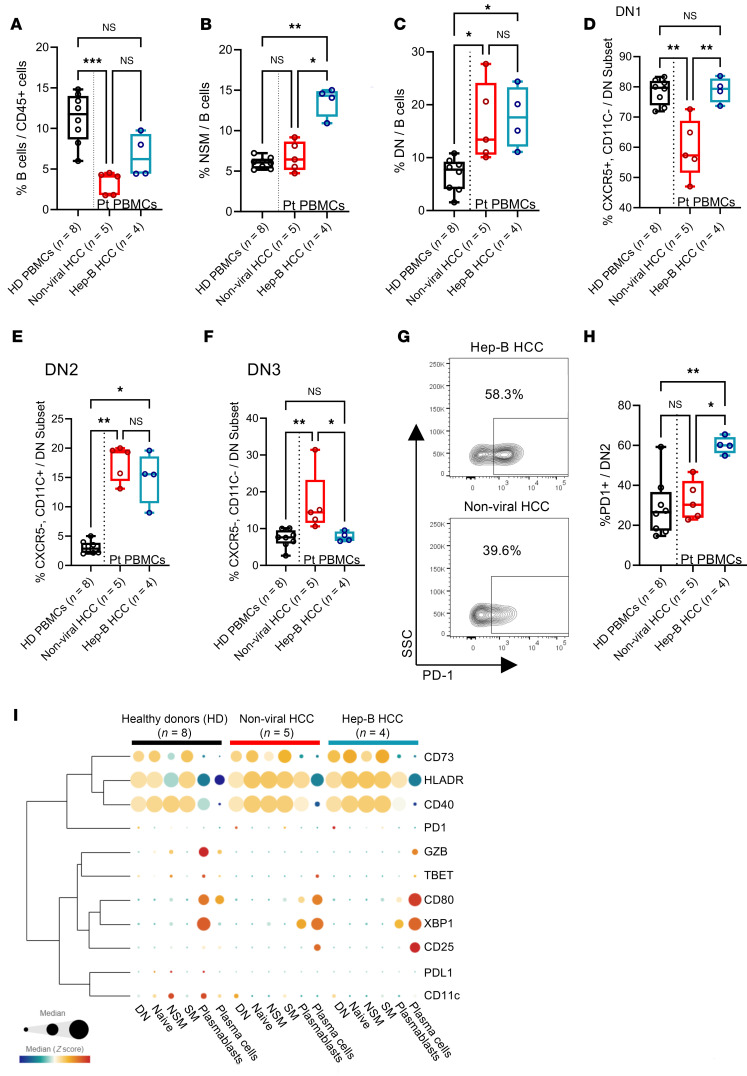
Expansion of DN2 and nonswitched memory B cells in peripheral blood of HCC patients. (**A**) Percentage of total B cells versus CD45^+^ cells within peripheral blood of healthy donors compared to nonviral and viral HCC patients. HD, healthy donor. Frequencies of (**B**) nonswitched memory (NSM) B cells and (**C**) DN (CD27^–^IgD^–^) B cells versus total B cells within peripheral blood of healthy donors compared to nonviral and viral HCC patients. Frequencies of (**D**) DN1, (**E**) DN2, and (**F**) DN3 subsets within the DN B cell population in peripheral blood of healthy donors compared to nonviral and viral HCC patients. (**G**) Representative flow cytometry dot plot (left) and (**H**) percentage of PD-1^+^ cells within the DN2 B subset in the peripheral blood of healthy donors compared to nonviral and viral HCC patients (right). (**I**) Bubble dot plot for various functional markers expressed on B and plasma cell subsets within the peripheral blood of healthy donors and HCC patients. One-way ANOVA with multiple comparisons using Fisher’s LSD test was used to test for statistical significance within each B cell subset (also refer to Supplemental Figure 7, F–H). Kruskal-Wallis test was used for significance testing (**A**–**H**). *P < 0.05; **P < 0.01; ***P < 0.001. NS, not significant. Sample sizes reported in labels of all graphs and heatmaps.

Finally, we sought to further identify B cell phenotypes within the peripheral blood of HCC patients that would be distinct from those of healthy individuals using the same flow cytometry approach (Supplemental Figure 8A). We first observed that the frequency of total circulating B cells was significantly decreased only in nonviral HCC patients (Figure 6A). Comparing peripheral blood mononuclear cells (PBMCs) from HCC patients to healthy donors, significantly higher frequencies of NSM B cells were found in HBV-driven HCC cases despite a trend that nonviral cases also had slightly higher proportions of these cells (Figure 6B). Compared with healthy donor PBMCs, we found higher proportions of DN (CD27^–^IgD^–^) memory B cells in HCC patient PBMCs (Figure 6C). Other defined B cell subsets did not show any significant differences between different sample groups (Supplemental Figure 8, B–D). Within peripheral blood of HCC patients, CXCR5^+^ DN1 B cells were reduced in nonviral cases, while the DN2 subset was significantly increased in both HBV-related and nonviral cases (Figure 6, D–F). At the same time, these DN2 cells had higher expression of XBP1 and PD-1 (Supplemental Figure 8E). Of interest, these DN2 B cells were previously known to resemble ABCs for their immunoregulatory functions in autoimmune diseases (24, 26). While chronic hepatitis studies found atypical CD27^–^TBET^+^ memory B cells to highly express PD-1 (22, 28), we observed here that PD-1 expression of peripheral DN2 B cells was increased only in HBV-HCC (Figure 6, G and H). In contrast, PD-1 and TBET expression seemed to be higher in DN3 B cells than in DN2 B cells. Phenotypic similarities between blood and tumor DN B cells were the higher expression of CD40 in DN1 B cells and XBP1 in DN2 cells (Supplemental Figure 8E).

Further profiling of functional markers in other B cell subsets revealed increased frequencies of CD73^+^ NSM B cells and CD25^+^ plasma cells in HCC samples (Figure 6I and Supplemental Figure 8, F and G), suggesting their potential identity as peripheral Bregs. Notably, we observed CD25 and spliced XBP1 (marker for ER stress response) to be downregulated within plasmablasts, while conversely, they were both upregulated in plasma cells of HCC patients (Figure 6I and Supplemental Figure 8, G and H). Taken together, our analysis presented several HCC-associated B cell phenotypes that are not limited to the TME but rather they could be identified within the peripheral blood, potentially as traceable blood biomarkers following HCC progression.

## Discussion

Immune profiling of virus-driven cancers may reveal critical mechanistic crosstalk between virus-mediated and tumor-induced adaptive immunity. Oliviero et al. demonstrated that HBV and HCV infections differentially influenced B cell activation to enhance their differentiation and reduce their proliferative capacity (29). On the other hand, later studies found that chronic HBV infections led to B cell dysfunction in terms of BCR signaling, antibody production, and an accumulation of an atypical CD27^–^PD-1^+^ memory B cell phenotype (22, 28). Interestingly, targeting PD-1 could partially restore antibody production in chronic HBV infections (22, 28), likely enhancing immunity similar to immune checkpoint blockade in HCC. More importantly, these studies found that the effects of chronic hepatitis were not limited to virus-specific B cells (28, 29). While the biology of B cells is indeed well characterized in chronic hepatitis, our present study explored the potential global B cell changes in HCC by transcriptome and flow cytometric profiling of TIL-B and plasma cells in liver tissues and peripheral blood of HCC patients. From both our cohort and a publicly available dataset, we identified downregulation of genes related to BCR signaling within B cells found in virus-associated HCC tissues. Furthermore, a proportion of plasma cells and TNF^+^ memory B cells were found to downregulate these BCR-signaling genes within both non-TMEs and TMEs. Our present study also employed patient-derived HCC organoids to mimic the TME in vitro, demonstrating that HCC-conditioned B cells would have a dampened response to BCR restimulation and suppressed production of antibodies. Additionally, B cells upregulated TBET and IL-10 production in these organoid cultures to acquire a Breg phenotype.

While the regulatory role of plasma cells in HCC was previously studied, we focused on the *TNF^+^* memory B cell subset that was also enriched for inflammatory pathways, sharing a distinct gene signature as a subset of B cells associated with immune tolerance from a different study cohort of patients receiving anti–PD-1 therapy (17). At present, the role of B cell–derived TNF-α within the TME remained elusive even though it was earlier demonstrated to contribute to squamous carcinogenesis (30). Interestingly, TNF-α upregulation was found to precede IL-10 production during Breg activation (31). Our current understanding is that Bregs might have similar roles in immune tolerance across various cancers and autoimmune diseases (20). Over a decade ago, IL-10–producing Bregs were reported to correlate with hepatic flare in patients with chronic hepatitis B (7). Another study by Xiao et al. also identified a subset of PD-1^hi^ B cells that correlate with disease stage and HCC recurrence. Upon activation, these PD-1^hi^ B cells produced IL-10 to suppress antitumor immunity (5). Although IL-10 production is the most recognized feature of Breg functionality, Glass et al. demonstrated that IL-10–producing B cells do not have a unique phenotype but could instead represent a transient activation state to drive immune tolerance (31). There is therefore a rationale to further explore alternative functional markers that could be associated with the Breg phenotype. Within the tissue microenvironment, our present study identified NSM and SM B cells that expressed PD-L1, which likely function as Bregs within the TME. While we anticipated that the DN atypical memory B cells would have an ABC-like (CD11C^+^TBET^+^) phenotype, our analysis revealed that a larger proportion of NSM B cells were CD11c^+^TBET^+^ double positive. The accumulation of ABC-like cells was observed in patients with chronic HCV infection but their functions were not determined (23). We speculated that these NSM B cells might play a role within the HCC TME to suppress antitumor responses. While the functional implications of these “ABC-like” NSM cells remains to be elucidated, we found these B cells to highly express CD25 and produced granzyme B that was previously demonstrated to be involved in Breg-mediated eradication of T cells (25). Furthermore, we demonstrated in vitro that B cells could upregulate TBET and acquire a similar NSM Breg phenotype to suppress cytotoxic CD8^+^ cells. Of note, a previous study showed that tumor-exposed Bregs have a similar CD25^hi^ phenotype and were capable of inducing TGF-β–dependent conversion of resting CD4^+^ T cells to FoxP3^+^ Tregs to support breast cancer metastasis (32). Likewise, Breg-mediated suppression of T and NK cells could be reversed by TGF-β or PD-L1 inhibition in the context of breast cancer (12).

It is plausible that Bregs could also be found within peripheral blood of HCC patients, considering that a recent report that found patients with HCV-HCC to have significantly higher frequencies of circulating IL-10^+^ Bregs compared with chronic HCV–infected patients or healthy controls (33). While Bregs were triggered by inflammation and autoantigen-mediated activation (34), the expansion of TBET^hi/+^ B cells was also reported in both chronic HBV and HCV infections (22, 23). Here, we uncovered an expanded population of PD-1^+^ DN2 (CXCR5^–^CD11c^+^) memory B cells that also expressed higher levels of XBP1 and TBET in the peripheral blood of HCC patients. We postulated that the observed upregulation of PD-1 in DN2 B cells could share phenotypic homology with chronic HBV–associated atypical memory B cells (22, 28). Interestingly, similar TBET-driven DN2 B cells were previously recognized as immunoregulatory precursors of autoimmune plasmablasts found to be expanded in systemic lupus erythematosus and severe eosinophilic asthma (26, 35). At the same time, we also uncovered increased CD73^+^ NSM B cells and CD25^+^ plasma cells within peripheral blood of HCC patients.

The main limitation of the present study was that the local demographics of our study cohorts that comprised mostly elderly patients and dominated by HBV-associated HCC (18). Hence, it remains to be determined whether age or viral history are drivers of ABCs in HCC. Our study is also limited in scope as we focused on characterizing the diverse B cell profiles in HCC tissues and blood. Several molecular targets were identified to be relevant in the understanding of Bregs and their role in immune tolerance but warrant further validation that would be more feasible as separate follow-up studies. While the use of organoids has been reported for the study of HBV replication and drug screening (36), our organoid coculture experiments would not be sufficiently robust to further address how hepatitis chronically modulates B cells during tumor progression. To date, the biology of B cells in viral HCC is largely understood based on human centric studies. While immunoregulatory roles of B and plasma cells were mostly demonstrated in nonviral HCC or MASH-HCC in vivo (5, 6), there are no reported studies to demonstrate how hepatitis virus influences the immune landscape of HCC in mouse tumor models. The integration of the viral genome to develop transgenic HBV or HCV mice models was reported to cause hepatocarcinogenesis (37, 38). However, HCC development in such transgenic mice could take up to 18 months (37). Future studies would have to thoroughly evaluate the feasibility and relevance of these mouse models for the purpose of studying immune regulation.

To the best of our knowledge, there is currently no established link between cancer and ABC, even considering the complex plasticity and heterogeneity of overlapping human B cell phenotypes (14). Recently, Ma et al. revealed the presence of similar ABC-like B cells in the TME of various cancers (21), further validating the findings of our current study. Nonetheless, there remains an unresolved conundrum in whether CD11c^+^TBET^+^ B cells are truly functional ABCs or rather an atypical phenotype of tissue-like memory B cells, as previously reported and discussed in chronic viral infections (39). Considering the potential influence of humoral immunity on the effectiveness of cancer immunotherapy, future studies could expand on HCC cases of nonviral and HCV etiologies to further characterize how the TME could influence virus-associated B cell phenotypes. Indeed, understanding the role and function of B cells in virus-driven cancers could potentially open up novel therapeutic opportunities against HCC.

## Methods

Further reagent details apart from flow cytometry antibodies are summarized in Supplemental Table 5.

### Sex as a biological variable.

Both male and female patients were included, and sex was not considered as a biological variable. PBMCs from healthy donors were deidentified and anonymous.

### Tissue dissociation for single-cell RNA-seq workflow.

Surgical resection of treatment-naive HCC samples was used for single-cell sequencing studies. Prewarmed Dulbecco’s modified Eagle medium (DMEM, Thermo Fisher Scientific) was used to rinse the tissue twice before further single-cell dissociation. Samples were minced manually by scalpel and transferred to 50 mL Falcon tubes. In total, 5–20 mL of dissociation buffer, containing DMEM, Collagenase P (Roche), and RNase-free DNase I (Thermo Fisher Scientific) was used depending on the size of tissue samples. The samples were digested at 37°C with agitation for 10 minutes. Single-cell suspensions were then filtered through a 70 μm filter, followed by a 40 μm filter. Red blood cell (RBC) lysis was performed using RBC Lysis Solution (Miltenyi Biotec) at room temperature for 10 minutes. The RBC-free single-cell suspension was subsequently washed 3 times with ice-cold 1% BSA and centrifuged at 300*g* for 10 minutes for the removal of debris and dead cells. Viability of all samples was validated to be in the range of 70%–95% before consideration for downstream single-cell sequencing. Cell suspension was finally resuspended in ice-cold 0.5% BSA for single-cell RNA-seq (scRNA-seq) workflow. Of note, prior testing found that this dissociation method (collagenase + DNase I) yields slightly higher recovery of macrophages and B cell populations for scRNA-seq analysis (data not shown).

### scRNA-seq.

The scRNA-seq workflow was carried out using the Chromium Next GEM Single Cell 5′ (v2 Chemistry Dual Index) platform with a target of approximately 10,000 cells (10× Genomics). Briefly, 1,000 cells/μL were added onto a well of Chip K to form gel bead-in-emulsion (GEMs) in the 10× Chromium instrument, followed by cell lysis, cDNA conversion, barcoding, fragmentation, adaptor ligation, and addition of sample index to the libraries before sequencing as per the manufacturer’s instructions. Sequencing of the pooled libraries with PhiX spike-in was done on S4 lanes of a NovaSeq instrument by Macrogen SG. A sequencing depth of 50,000 reads/cell was targeted for each sample. The standard CellRanger v6.0 (10× Genomics) analysis pipeline was used to filter gene expression matrix files. The files of filtered_feature_bc_matrix.h5 were then imported into Partek Flow for downstream analysis. Doublets were removed using standardized single cell QA/QC procedure. Cells with either a mitochondrial percentage greater than 25% or with number of detected genes more than 6,000 or read counts of more than 35,000 were excluded as potential doublets. Additionally, filtered cells were exported as an h5ad file to further cross-check for putative doublets using Scrublet package (40). After filtering, we obtained a total of 85,607 high-quality single cells, ranging from 1,829 cells to 11,035 cells per sample for subsequent log_2_(CPM + 1) normalization, principal component analysis (PCA), and t-distributed stochastic neighbor embedding (tSNE)/uniform manifold approximation and projection (UMAP) analysis.

*MS4A1*^+^ B cells and *MZB1*^+^ plasma cells were manually selected from the tSNE and UMAP plots of the concatenated single-cell data (cell counts provided in Supplemental Table 3). Subclustering was first performed on 2,810 selected B and plasma cells, followed by Louvain graph–based classification. The clusters were further manually refined by serial analyses of DEGs, and re-clustering. The gene count matrix was downloaded from the NCBI GEO (GSE149614) (15) and analyzed using the Scanpy package (v1.9.1) (https://scanpy.readthedocs.io/en/stable/). After data filtering and log normalization, dimensionality reduction using PCA was performed, followed by cell clustering using the Leiden algorithm. The BCR score was calculated (scanpy.tl.score_genes) using the BIOCARTA_BCR_PATHWAY signature from GSEA (41). The anti–PD-1 cohort dataset was accessed from http://biokey.lambrechtslab.org/ Using the B cells annotated by the original authors, we performed similar Partek pipelines for differential gene expression analysis as described above.

Next, we analyzed the regulon activity for the scRNA-seq data using SCENIC v0.12.1. The pipeline involves 3 steps: (i) Coexpressed sets of genes and transcription factors were identified using the GRNBoost2 algorithm. (ii) Regulon prediction based on *cis*-regulatory motif analyses was then performed using cisTarget. (iii) AUCell was employed to score the activity of each regulon in the cells. A total of 262 regulons were identified from this analysis. Regulon specificity scores (RSS) were calculated for clusters identified based on gene expression data using the regulon_specificity_scores function from the pySCENIC pipeline with default parameters. Number of shared regulons between clusters was computed after filtering the RSS scores by 0.35. The cutoff was selected to include the approximately top 50% of the data.

### BCR sequencing.

The 10× Genomics CellRanger VDJ analysis workflow was used to analyze the immune repertoire of B cell data. The raw reads were aligned to the human reference genome GRCh38 shipped with CellRanger VDJ (GRCh38-alts-ensembl-7.1.0). Using the aligned reads, CellRanger VDJ identified and assembled V(D)J sequences for determining the variable (V), diversity (D), and joining (J) genes that make up the immune receptor sequences. The complementarity-determining region 3 (CDR3) was also detected. Artifacts, including low-quality sequences, PCR duplicates, and nonproductive rearrangements, were filtered using the default settings. BCR clonotypes were then identified through performing clustering on CDR3 sequences (scRepertoire R package, v1.4.0) (https://www.bioconductor.org/packages/release/bioc/html/scRepertoire.html). Within each CDR3 cluster, CDR3 sequences that are identical or have default level of similarity were regarded as unique clonotypes, i.e., BCR clones. Cell barcodes were assigned to the clonotypes, and only clonotypes with cell barcodes also found in the 5 B cell clusters defined by scRNA-seq analysis were included in the alluvial visualization (alluvialClonotypes in scRepertoire), where the clonotypes were colored by B cell clusters defined by scRNA-seq analysis and clonotype proportions were stratified by tumor status, viral status, and B cell clusters.

### Application of gene signature for TCGA analysis.

Gene expression and clinicopathological data of the liver cancer cohort from TCGA was accessed from UCSC Xena (https://xenabrowser.net/). The gene signature (*ZBTB16*, *TXNIP*, *STAG3*, *TCL1A*, *AFF3*, *HLA-DMB*, *FAM177B*, *IFITM1*, *RPL36A*, *RPS10*, *HSPA1B*, *IFI44L*, *HLA-DQA2*, *XAF1*) was derived from common upregulated DEGs expressed in B cell cluster 1 from our single-cell transcriptomics analysis. *CEMIP2* and *FCMR* were excluded due to data unavailability within the TCGA dataset. Only tumor samples were used for immune deconvolution and GSEA.

### In vitro tumor cell–B cell cocultures.

Healthy donor–derived PBMCs were used for the isolation of B cells for the in vitro assays. In brief, the leukocyte fraction from blood donors was processed using Ficoll-based density gradient centrifugation to obtain PBMCs. Subsequently, B cells were isolated by negative selection using a commercially available magnetic bead–based isolation kit (Miltenyi Biotec). Purified B cells were maintained in AIM-V media (Thermo Fisher Scientific) supplemented with 100 IU/mL of IL-2 and 30 ng/mL of IL-4 (Miltenyi Biotec). HCC tumor cell lines HUH-7 (JCRB Cell Bank) and SNU-475 (ATCC) were maintained in 10% FBS in RPMI 1640 media (Thermo Fisher Scientific). Adherent tumor cells were cultured with B cells at a 1:5 ratio. After 2 days of culturing, B cells were either sorted for FACS phenotyping or for subsequent T cell costimulation assay.

### Generation of patient-derived organoids and B cell coculture.

Using up to 300,000 live cells from patient tissue dissociation, either nontumor or tumor cell suspensions were seeded in 24-well plates in a hydrogel dome using Geltrex LDEV-Free Reduced Growth Factor Basement Membrane Matrix (Thermo Fisher Scientific). Next, organoid formation was carried out using an organoid initiation protocol from a commercial organoid culture kit (Hepaticult, STEMCELL Technologies). Propagation and differentiation were also carried out using the organoid growth medium and differentiation medium accordingly from the same kit. Allogeneic B cells were cocultured with organoids in an approximate cell count ratio of 5:1 (B cells/organoid cells) for 72 hours in RPMI 1640 media with 10% FBS. Subsequently, supernatants from these cocultures were collected for ELISA, while cells were harvested and processed for flow cytometry phenotyping and B cell restimulation and PhosFlow assay.

For 3D immunofluorescence and confocal imaging of tumor markers, organoids were fixed with 4% paraformaldehyde (Sigma-Aldrich) for 1 hour at 4°C. Subsequently, fixed organoids were processed using a MACS clearing kit (Miltenyi Biotec) for downstream processing and staining with rabbit anti-GPC3 primary antibody (Abcam, ab95363, clone SP86), goat anti–rabbit IgG (H+L) secondary antibody conjugated with Alexa Fluor Plus 488 (Thermo Fisher Scientific), and NucBlue Fixed Cell ReadyProbes Reagent (Thermo Fisher Scientific).

### IgG and IgM quantification by ELISA.

ELISAs were carried out on Maxisorp plates (Thermo Fisher Scientific) coated overnight at 4°C with IgG (Southern Biotech) and IgM (Southern Biotech). Plates were washed with 0.05% Tween 20 in PBS and blocked with 1% BSA for 1 hour. The plates were washed again and incubated with cell culture supernatants and their respective standards (Southern Biotech), diluted in 1% BSA, for 2 hours. Plates were then washed and incubated with corresponding secondary antibodies (Southern Biotech) for 1 hour. The plates were subsequently washed and TMB substrate (Life Technologies) was added to allow colorimetric development for no longer than 30 minutes. Finally, stop solution (1N HCl) was added and the absorbances were read using TECAN Infinite M200 with excitation at 450 nm and correction at 570 nm.

### Flow cytometry.

Patient PBMCs and tissue samples were obtained from a separate cohort (Supplemental Table 2). Here, we used a gentler tissue dissociation protocol with a kit (Miltenyi Biotec) that was validated to better preserve essential cell surface epitopes for flow cytometric analysis. Cells were subsequently washed with FACS buffer (5% FBS in PBS) and intracellular FACS staining was performed using Transcription Factor Staining Buffer set (eBioscience). Antibodies used in flow cytometry are listed in Supplemental Table 4. For IL-10 staining, single-cell suspensions were treated with Brefeldin A (GolgiPlug, BD Biosciences) and monensin (GolgiStop, BD Biosciences) overnight according to the manufacturer’s protocol. Subsequently, surface and intracellular staining protocols were carried out using anti-CD45, anti-CD19, and PE-conjugated anti–IL-10 (clone JES3-19F1, BD Biosciences).

FACS acquisition was either done on a Novocyte Penteon (Agilent) or FACSymphony A3 cell analyzer (BD Biosciences). FACS isolation of B cells was performed using a FACSAria II system (BD Biosciences) after tumor coculture using fixable blue viability dye and Alexa Fluor 700–conjugated anti-CD45 (BD Biosciences). All FACS analysis was done using Flowjo software (BD Biosciences).

### B cell restimulation and PhosFlow assay.

After 3 days of coculture with patient-derived organoids, cells were dissociated from these 3D cultures using TrypLE express enzyme and resuspended in RPMI 1640 media for anti-IgM (Jackson Immunoresearch Laboratories) restimulation for 10 minutes. Subsequently, cell suspensions were fixed in Fix Buffer I (BD Biosciences) and permeabilized with Perm Buffer III (BD Biosciences) for downstream staining with PE-conjugated antibody targeting p-ZAP70/SYK (clone n3kobu5, Thermo Fisher Scientific) together with anti-CD19 and anti-CD45. Samples were acquired on a Novocyte Penteon (Agilent).

### T cell costimulation assay.

Prior to assay, B cells were cocultured with either HUH-7 or SNU-475 cells as mentioned above. Only cells in suspension were harvested for FACS, while remaining adherent tumor cells were discarded. Approximately 69.9% to 93.6% of recovered cells were viable CD45^+^ cells that were sorted for subsequent downstream assay. Of note, the control group that was not cultured with tumor cells was also subjected to the same FACS procedures (gating strategy and percentage of CD45^+^ cells are presented in Supplemental Figure 7, F and G).

Coculturing of FACS-isolated B and T cells was performed in an autologous setting. From matched donors, T cells were purified by negative selection using a T cell isolation kit (Miltenyi Biotec). In the presence of either tumor-experienced B cells or control B cells, CFSE-labelled T cells were cultured with CD3/CD28-stimulating Dynabeads (Thermo Fisher Scientific) supplemented with 100 IU/mL of IL-2. Cocultures of B and T cells were incubated for 3 days at a cellular ratio of 1:10, respectively. Inhibitors used included atezolizumab (MedChemExpress, 10 μg/mL) and anti–IL-10 neutralizing antibody (Thermo Fisher Scientific).

### Statistics.

Unless stated otherwise, all graphical presentation and significance testing was performed using GraphPad Prism. Wherever possible, all individual data points and sample sizes are shown together with either violin or box-and-whisker plots. Bounds of the boxes extends from the 25th to 75th percentiles. The line in the middle of the box represents the median while whiskers go down to the smallest value and up to the largest. Data points were excluded in the case of extreme outliers, poor sample viability, or inadequate B cell counts. Appropriate statistical tests are stated in figure legends. A *P* value of less than 0.05 was considered as significant. Data from flow cytometry are presented in Tukey box-and-whisker plots. All error bars represent standard deviation (SD) of the mean.

### Study approval.

PBMCs were isolated from deidentified human blood collected in accordance with and under the Singapore government’s Health Sciences Authority (HSA) residual blood project (ref. no. 201306-04). Patients diagnosed with resectable HCC were prospectively recruited at the Singapore General Hospital (SGH) before curative-intent surgery with written informed consent and approval from the Central Institution Review Board (CIRB ref. 2019/2303). All clinicopathological features of surgical resections were confirmed by the pathology department prior to the release of samples for research purposes. Characteristics of patient cohorts for single-cell sequencing and flow cytometry can be found in Supplemental Tables 1 and 2, respectively.

### Data availability.

The gene expression matrices (h5 files) and BCR sequencing data (BCRobj.RDS) have been made available in the Zenodo database (https://zenodo.org/records/14842263) Values for all data points in graphs are reported in the Supporting Data Values file. Other resources and alternative file formats of datasets used and/or analyzed during the current study are available from the corresponding author upon reasonable request.

## Author contributions

SYN, TWHS, SX, HCT, and KPL conceptualized the study. CT, RB, JL, HSC, HMC, CYC, AYFC, PCC, PRJ, JYT, YXK, AYC, PKHC, BG, WKW, WQL, TJZL, PYT, JK, NTN, and TKHL were involved in sample acquisition. SYN, TWHS, J. Chung, LC, JZ, and KL performed the experiments. SYN, TWHS, SK, ML, NS, J. Chung, J. Chua, and RD contributed to data analysis. SX, RD, HCT, and KPL were involved in the funding acquisition. All authors contributed to the writing of the manuscript.

## Supplementary Material

Supplemental data

Supporting data values

## Version 1 02/25/2025

In-Press Preview

## Version 2 04/08/2025

Electronic publication
