# Supplementary material for: Atypical memory B cells acquire Breg phenotypes in hepatocellular carcinoma
Source: JCI Insight. 2025 Feb 25;10(7):e187025. doi: 10.1172/jci.insight.187025 (PMC11981623; doi:10.1172/jci.insight.187025)
Supplement: Supplemental data [file jciinsight-10-187025-s159.pdf]

**Supplementary Table S1. Patient characteristics for single cell Transcriptomics and BCR sequencing**				
**No.**	**Sample ID**	**Hep status**	**Disease Background**	**Tumour Stage**
1	HCC002	Non-viral	Not stated	pT1b Nx Mx
2	HCC004	Hep B	Cirrhosis	pT1b Nx Mx
3	HCC006	Non-viral	Not stated	pT1b Nx Mx
4	HCC008	Non-viral	Not stated	pT4 Nx Mx
5	HCC009	Hep B	Child's Pugh A, Cirrhosis	pT2 Nx Mx
6	HCC010	Non-viral	Child's Pugh B9, NASH, Liver Cirrhosis	At least pT1b NX MX
7	HCC011	Hep C	Child's Pugh A, HCV treated	pT1b Nx Mx
8	HCC001	Hep C	No cirrhosis	pT3 Nx Mx
9	HCC012	Hep B	No cirrhosis	pT1b N0 M0
10	HCC005	Non-viral	Cirrhosis	pT1b N0 M0

Hep: Hepatitis; Nx: lymph nodes not evaluated; Mx: Metastasis not measured.

**Supplementary Table S2. Patient characteristics for flow cytometry cohort**				
**No.**	**Patient code**	**Hep status**	**Disease Background**	**Tumour Stage**
1	HCC013	Hep B	Hep B	pT1b Nx Mx
2	HCC014	Hep C	Child's Pugh A5, Cirrhosis	pT2 Nx Mx
3	HCC015	Hep B	Child's Pugh A5, Cirrhosis	pT1b Nx Mx
4	HCC016	Hep B	Child's Pugh A5, Cirrhosis	pT2 Nx Mx
5	HCC017	Hep B	Hep B	pT1b Nx Mx
6	HCC018	Hep B	Child's Pugh A5, Cirrhosis NAFLD	pT2 Nx Mx
7	HCC019	Non-viral	NASH Cirrhosis	mpT4 Nx Mx
8	HCC020	Non-viral	Not stated	pT3 Nx Mx
9	HCC021	Non-viral	Child's Pugh A5, Cirrhosis	pT2 Nx Mx
10	HCC022	Non-viral	Background liver cirrhosis	pT2 Nx Mx
11	HCC023	Non-viral	Not stated	pT1b Nx Mx
12	HCC024	Non-viral	Not stated	pT3 Nx Mx
14	HCC025	Hep B	Fibrosis present	pT4 Nx Mx
15	HCC026	Hep B	Not stated	pT1b Nx Mx
16	HCC027	Hep B	Not stated	pT1a Nx Mx

Hep: Hepatitis; Nx: lymph nodes not evaluated; Mx: Metastasis not measured.

Supplementary Table S3. Frequencies of B cells obtained in single cell sequencing cohort.

No.	ID	Viral status	Tissue	Total B cells	Total filtered cells	% of total cells	% of CD45+ cells
1	001N	HCV	Non-tumour	328	4946	6.63%	9.13%
2	001T	HCV	Tumour	180	11035	1.63%	3.75%
3	002N	Non-viral	Non-tumour	13	1829	0.71%	0.87%
4	002T	Non-viral	Tumour	24	4015	0.60%	1.25%
5	004N	HBV	Non-tumour	93	2843	3.27%	4.59%
6	004T	HBV	Tumour	102	4980	2.05%	3.33%
7	005N	Non-viral	Non-tumour	245	5174	4.74%	8.08%
8	005T	Non-viral	Tumour	183	4941	3.70%	5.96%
9	006T	Non-viral	Tumour	30	4020	0.75%	0.99%
10	008N	Non-viral	Non-tumour	51	3474	1.47%	2.13%
11	008T	Non-viral	Tumour	464	5152	9.01%	16.11%
12	009N	HBV	Non-tumour	202	6941	2.91%	3.58%
13	009T	HBV	Tumour	110	4083	2.69%	4.04%
14	010T	Non-viral	Tumour	130	4802	2.71%	4.31%
15	011N	HCV	Non-tumour	274	4810	5.70%	6.93%
16	011T	HCV	Tumour	12	2762	0.43%	0.98%
17	012N	HBV	Non-tumour	102	5556	1.84%	2.30%
18	012T	HBV	Tumour	267	4244	6.29%	10.00%

Supplementary Table S4. List of Flow cytometry Antibodies

s/n	Markers	Clone	Fluorochrome	Company	Catalog no.
1	Live/Dead Blue		Blue	ThermoFisher	L23105
2	CD11c	B-ly6	BUV661	BD Biosciences	612967
3	CD3	UCHT1	AF700	BD Biosciences	557943
4	CD14	HCD14	AF700	Biolegend	325614
5	CD56	HCD56	AF700	Biolegend	318316
6	CD19	SJ25C1	APC-Cy7	eBioscience	47-0198-42
7	CD45	HI30	BUV737	BD Biosciences	568524
8	IgD	IA6-2	BV786	BD Biosciences	740997
9	CXCR5	RF8B2	BUV395	BD Biosciences	740266
10	CD73	AD2	BUV563	BD Biosciences	748585
11	CD40	5C3	BUV805	BD Biosciences	742028
12	CD80	L307.4	BV650	BD Biosciences	564158
13	CD27	M-T271	BB700	BD Biosciences	560612
14	CD25	M-A251	PE-Cy7	BD Biosciences	557741
15	HLA-DR	L243	BV510	BD Biosciences	563083
16	PD1	EH12.2H7	BB515	BD Biosciences	564494
17	PDL1	MIH1	BV480	BD Biosciences	746346
18	CD38	HIT2	PE-Cy5	Biolegend	303508
19	CD138	MI15	PE	BD Biosciences	552026
20	XBP-1	143F	AF647	Biolegend	647505
21	Granzyme B	GB11	BV421	BD Biosciences	563389
22	Nur77	1E10A15	AF594	Biolegend	653504
23	T-bet	O4-46	BV711	Biolegend	644820
24	SYK	4D10	FITC	BD Biosciences	552476
25	Brilliant stain buffer			BD Biosciences	586385

Supplementary Table S5. List of other reagents

s/n	Name of reagent/supplement	Brand	Catalogue number
1	AIM-V medium	Gibco	12055091
2	RPMI 1640 medium, HEPES	Gibco	22400071
3	Human IL-2 IS, research grade	Miltenyi Biotec	130-097-743
4	Human IL-4, research grade	Miltenyi Biotec	130-093-917
5	CFSE Cell Division Tracker Kit	Biolegend	423801
6	Dynabeads human T-activator CD3/CD28 for T cell expansion and activation	Gibco	11131D
7	Ateozolizumab	MedChemExpress	HY-P9904
8	Il-10 monoclonal antibody (JES3-9D7)	Invitrogen	AHC0103
9	Pan B cell isolation kit, human	Miltenyi Biotec	130-101-638
10	Pan T cell isolation kit, human	Miltenyi Biotec	130-096-535
11	Tumour dissociation kit, human	Miltenyi Biotec	130-095-929
12	Transcription Factor Staining Buffer Set	eBioscience™	00-5523-00
13	Geltrex LDEV-free reduced growth factor basement membrane matrix	Gibco	A1413202
14	HepatiCult Organoid Kit (Human)	Stem cell technologies	100-0386
15	AffiniPure F(ab’)2 Fragment Goat anti-human IgM	Jackson ImmunoResearch Laboratories	109-006-129
18	Goat anti-human IgM	Southern Biotech	2023-01
19	Human IgG for ELISA standards	Southern Biotech	0150-01
20	Human IgM Lambda for ELISA standards	Southern Biotech	0158L-01
21	Goat anti-human IgG-HRP	Southern Biotech	2045-05
22	Goat anti-human IgM-HRP	Southern Biotech	2023-05
23	TMB substrate	Life Technologies	SB02

Supplementary Figure S1

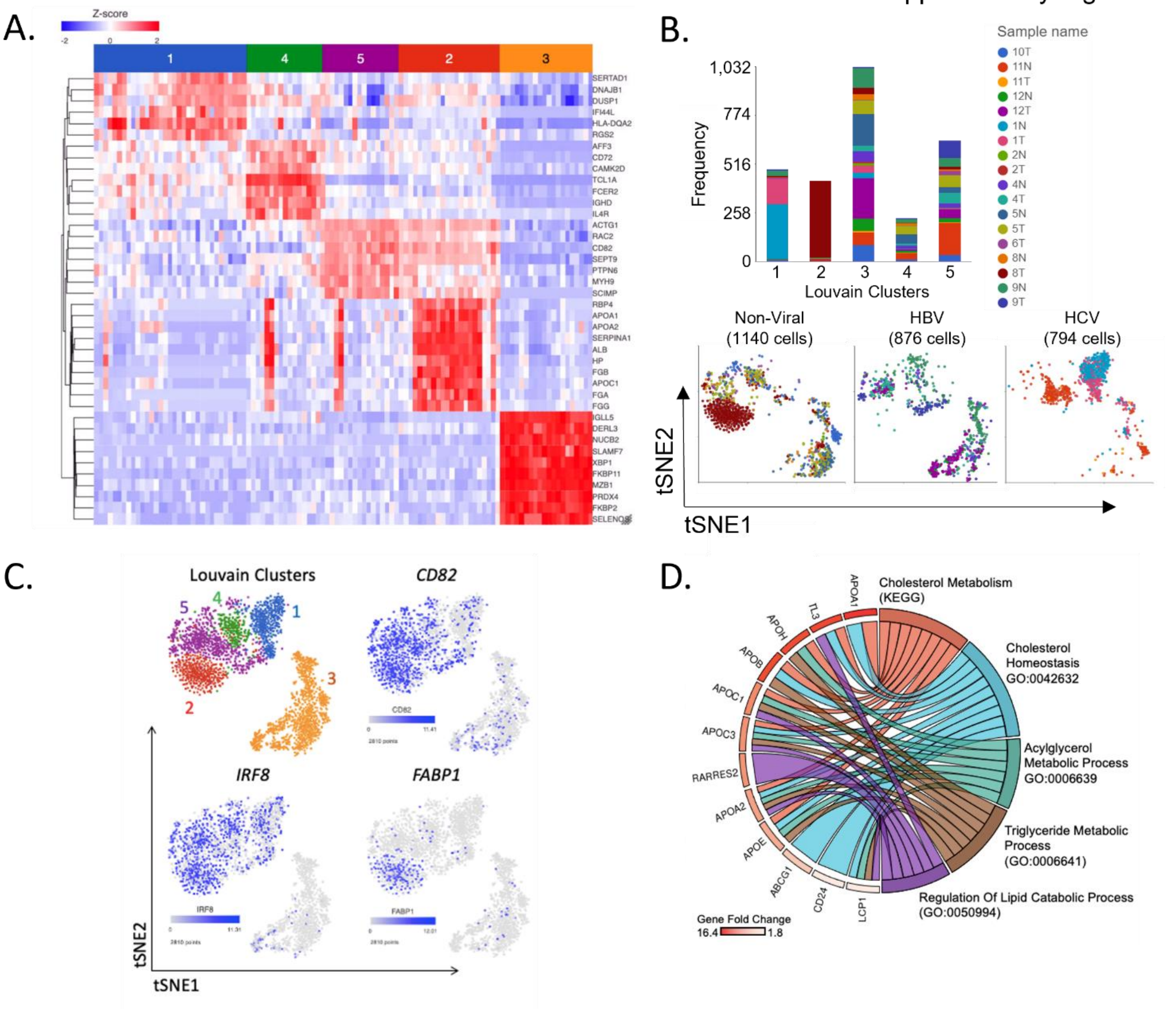

Supplementary Figure S1. Differentially expressed genes (DEGs) based on unsupervised clustering of B and plasma cell clusters in HCC patients.

(A) Top DEGs for each clusters are presented in the hierarchical heatmap clustering. **(B)** Distribution of HCC patient samples within the 4 clusters of B and 1 cluster of plasma cells as identified by Louvain clustering and visualised as **(B, top)** frequency bar plot **or (B, bottom)** tSNE analysis sub-divided based on viral status. **(C)** tSNE analysis revealing cluster 2 that co-expressed CD82, FABP1 and IRF8. **(D)** Chord diagram showing lipid related genes and the significantly enriched lipid gene sets in cluster 2.

Supplementary Figure S2

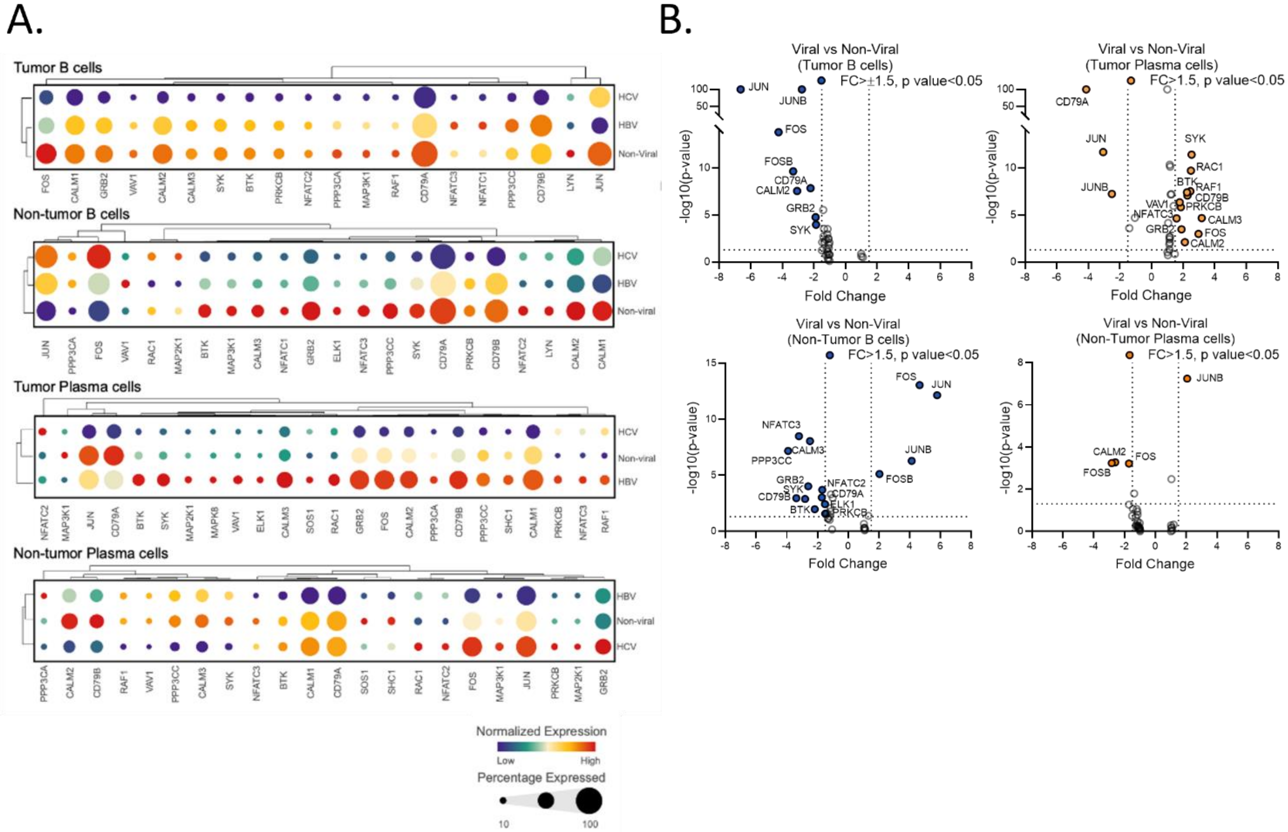

Supplementary Figure S2. BCR-related genes are differentially expressed in B cells and plasma cells of different viral status and tissue types.

(A) Bubble heatmap with unsupervised clustering showing BCR-related DEGs comparing B cells and plasma cells based on viral status in tumors and non-tumor samples. **(B)** Volcano plots showing significant BCR-related DEGs comparing viral-HCC (pool of HBV-HCC and HCV-HCC) to non-viral samples of tumors and non-tumors. Hurdle model was used to test for significance.

Supplementary Figure S3

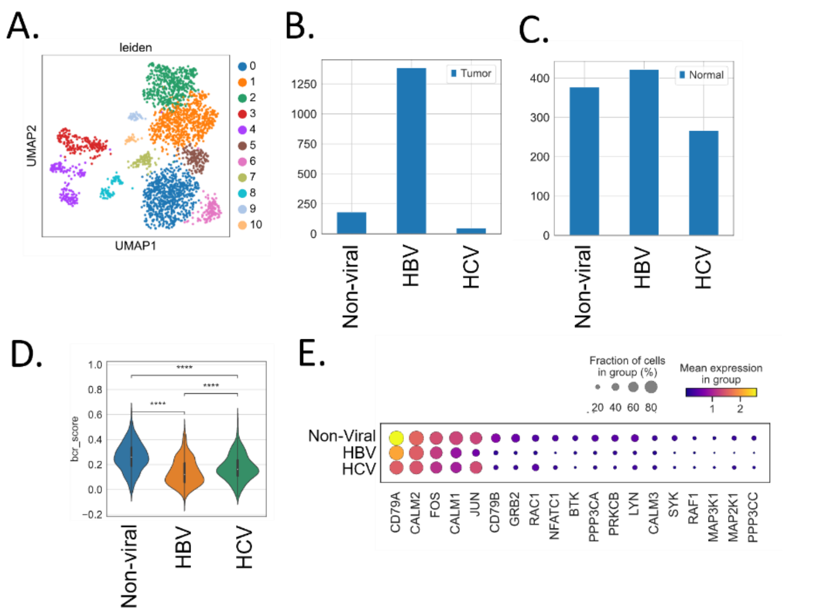

Supplementary Figure S3. Clustering and characterization of B cells from a publicly available HCC dataset (GSE149614).

(A) B cell clusters obtained from non-viral, HBV-HCC and HCV-HCC cohorts. Cell counts for the different cohorts from **(B)** tumor and **(C)** adjacent normal regions. **(D)** Overall BCR signalling pathway score and **(E)** average expression of individual genes, plotted for the adjacent normal samples. Only genes with mean expression >10% are shown.

Supplementary Figure S4

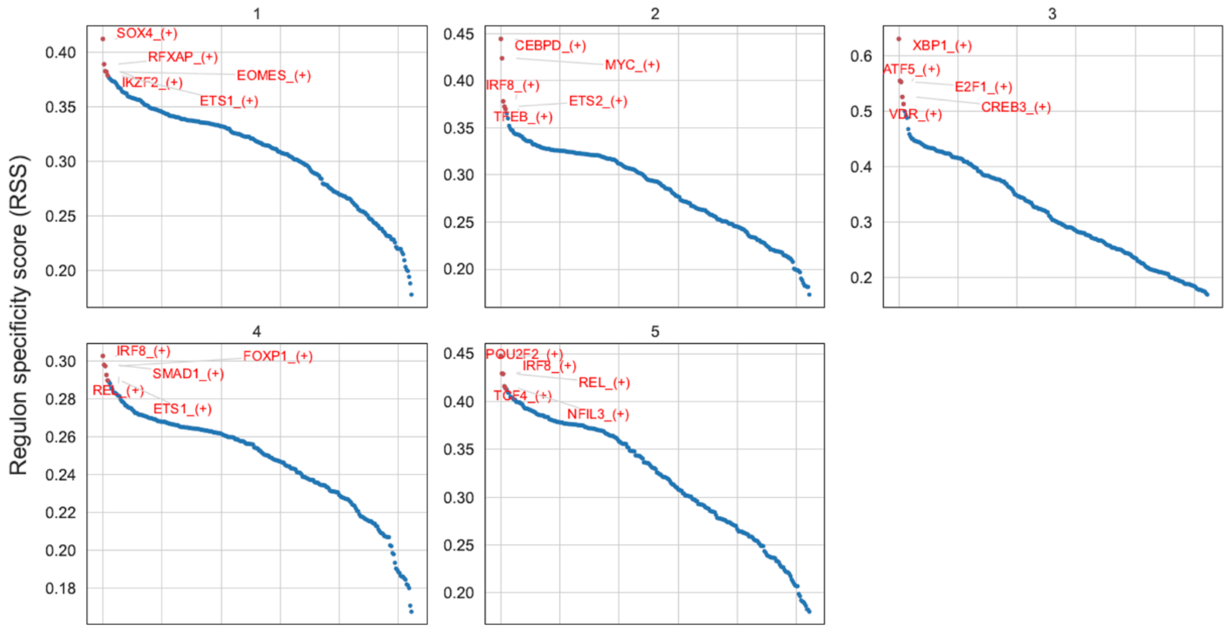

Supplementary Figure S4. **Regulon specificity scores (RSS) for clusters identified from the gene expression data.** Five panels represent five Louvain clusters identified based on the gene expression data. Top 5 regulons in each cluster are indicated in red.

Supplementary Figure S5

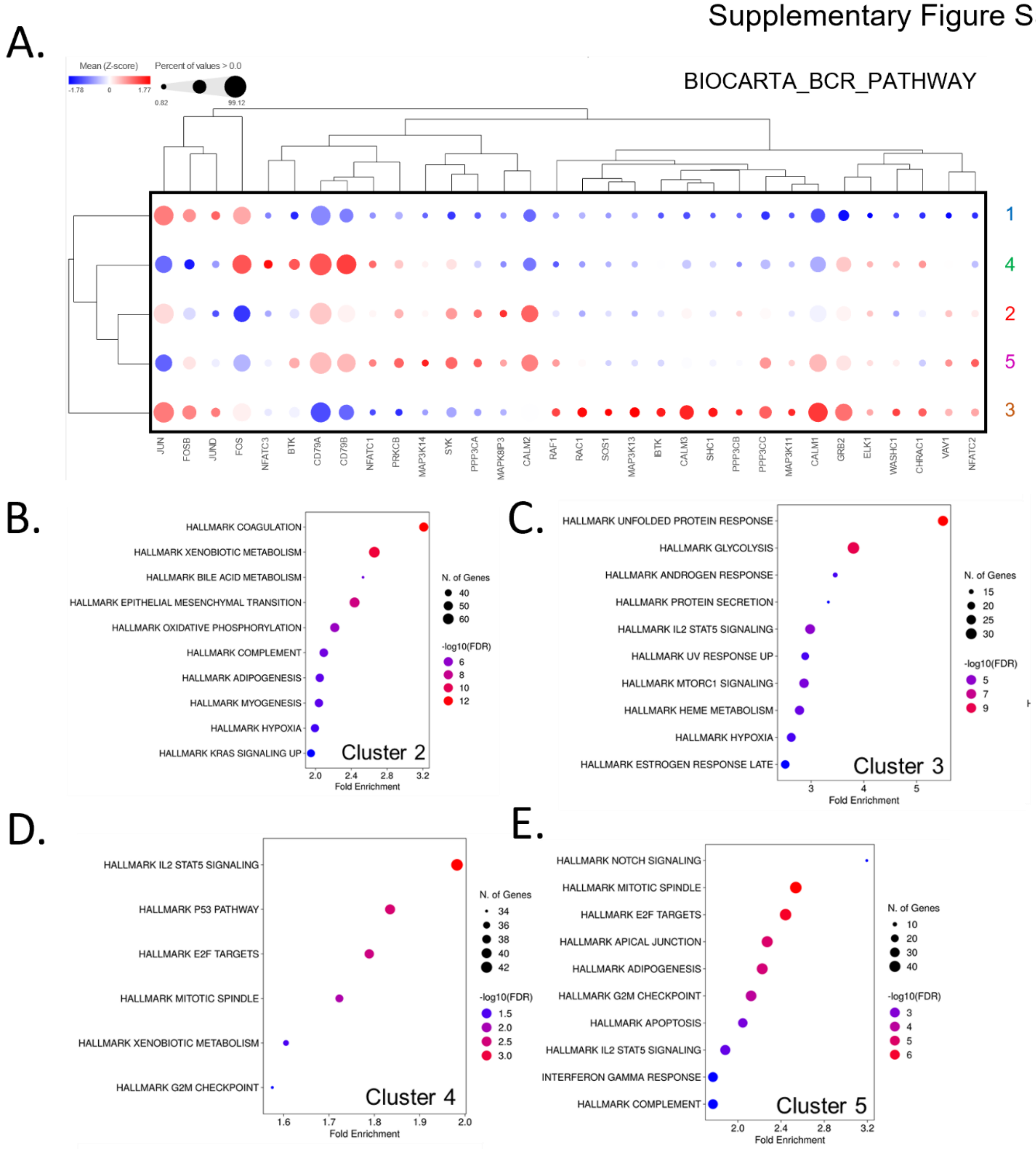

Supplementary Figure S5. Gene enrichment analysis of B cell subsets for hallmarks gene sets. **(A)** Bubble heatmap showing differentially expressed genes within the Biocarta BCR pathway gene set. The hurdle statistical model was used for differential gene expression analysis. **(B to E)** Gene enrichment was done based on top 200 differentially expressed genes with reference to MSigDB Hallmarks gene sets. Only top 10 significant gene sets are shown for each cluster.

Supplementary Figure S6

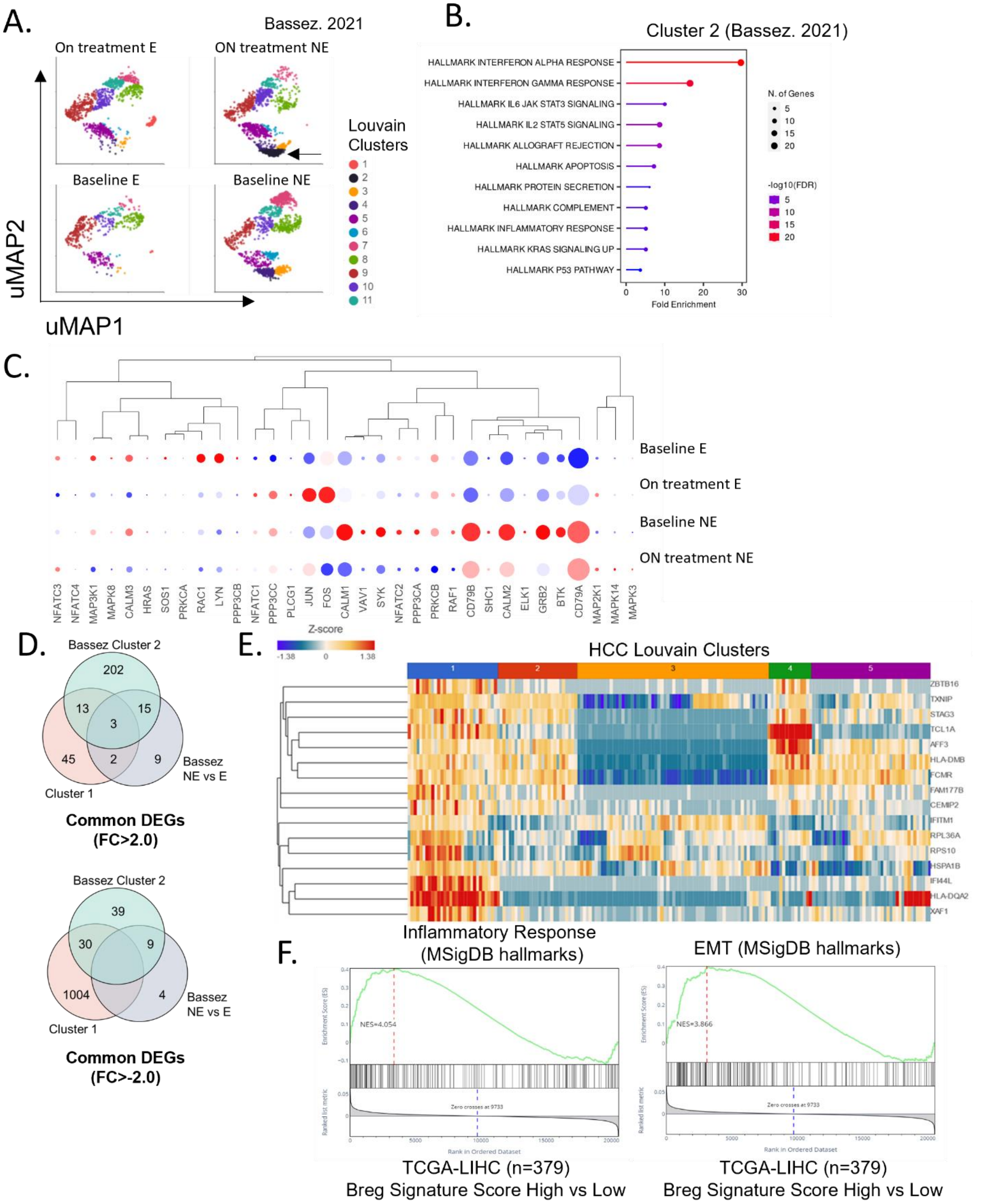

Supplementary Figure S6. Gene expression signature of Bregs associated with immune tolerance to anti-PD1 therapy

(A) UMAP projections of B cell subclusters found in breast cancer patients before and after treatment with anti-PD1 therapy. Plots were split based on sample type (baseline versus on-treatment) and patient's response to therapy (E: "Responders" with Clonal expansion of T cells upon therapy, NE: "Non-responders" with no clonal expansion of T cells upon therapy). **(B)** Gene enrichment was done for B cell cluster 2 (Bassez. 2021 Cohort) based on top 200 differentially expressed genes with reference to MSigDB Hallmarks gene sets. Only top 10 significant gene sets are shown. **(C)** Bubble heatmap with hierarchical clustering of BCR-related genes differentially expressed in tumoral B cells comparing baseline to on-treatment samples in responders ("E") and non-responders ("NE"). **(D)** Venn diagram showing number of overlapping DEGs in 3 different comparisons. Only significant DEGs with a fold change of more than ±2 was analysed. **(E)** Normalized expression heatmap for the 16 common upregulated genes associated with Breg phenotype among the 5 B and plasma cell clusters in HCC cohort. **(F)** GSEA analysis of TCGA liver cancer cohort (n=379 primary tumors). Tumors were classified into two groups based on the median of Breg signature score.

Supplementary Figure S7

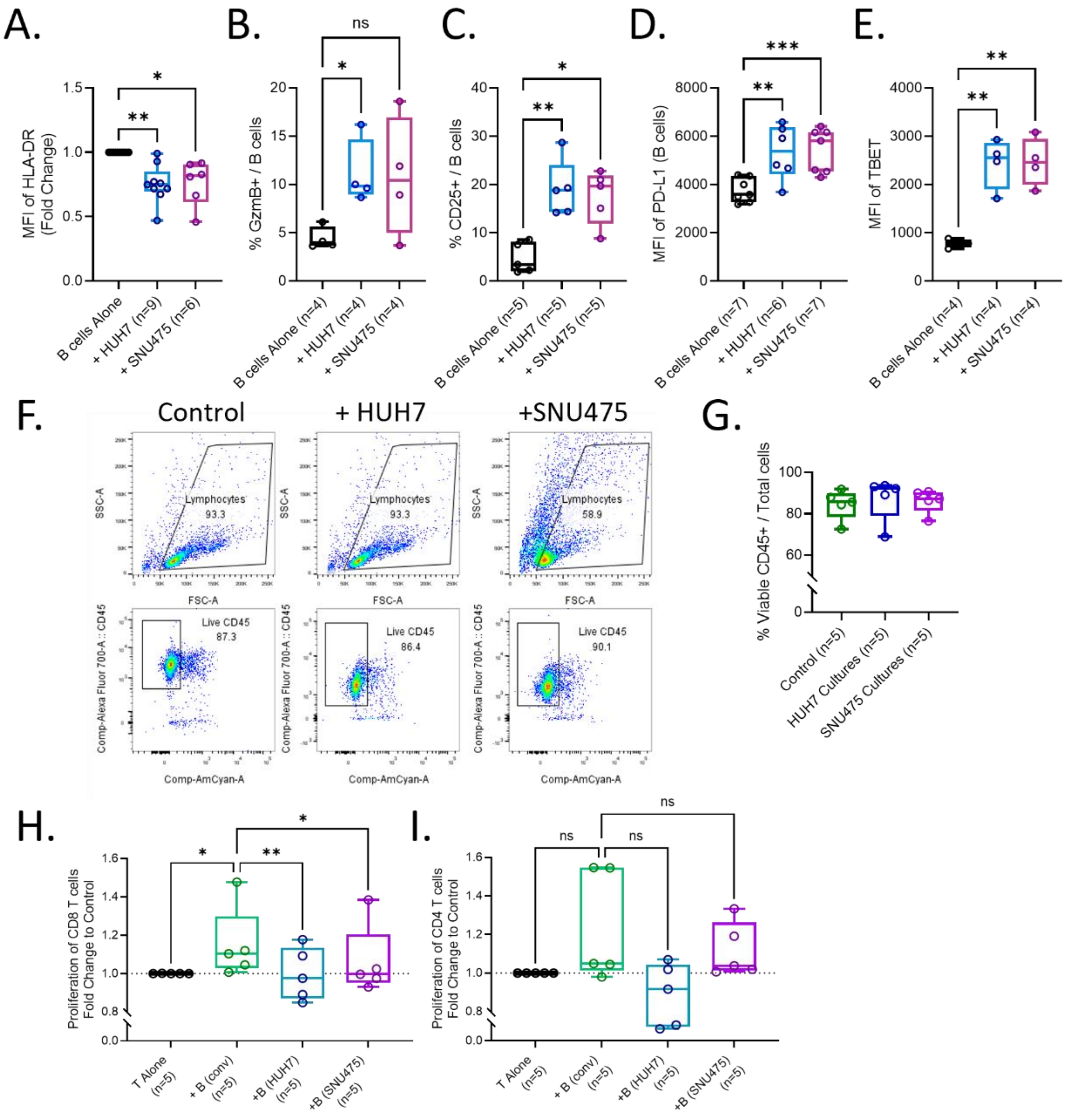

Supplementary Figure S7. B cells acquire regulatory phenotype to suppress CD8 T cell activation.

(A) Mean fluorescence intensity (MFI) of HLA-DR, expressed on B cells 3 days of co-culturing B cells with HCC tumor cell lines-HUH7 or SNU475. **(B)** Percentage of Granzyme B+ and **(C)** CD25+ B cells after 3 days of co-culturing B cells with HCC tumor cell lines-HUH7 or SNU475. **(D)** MFI of PD-L1 and **(E)** TBET expressed on B cells cells after 3 days of co-culturing B cells with HCC tumor cell lines-HUH7 or SNU475. **(F)** Representative flow cytometry dot plots for gating strategy used in FACS sorting (n=5 biological replicates). **(G)** Percentage of viable CD45+ cells sorted from all experimental conditions. **(H)** Proliferation of CD8 T cells and **(I)** CD4 T cells in the presence of differentially stimulated B cells as compared to T cell alone (Control). Proliferation index was normalized to control. **(H and I)** One-way ANOVA with multiple comparisons was used to test for significance. *<0.05, **<0.01, ***<0.001 and ns= non-significant.

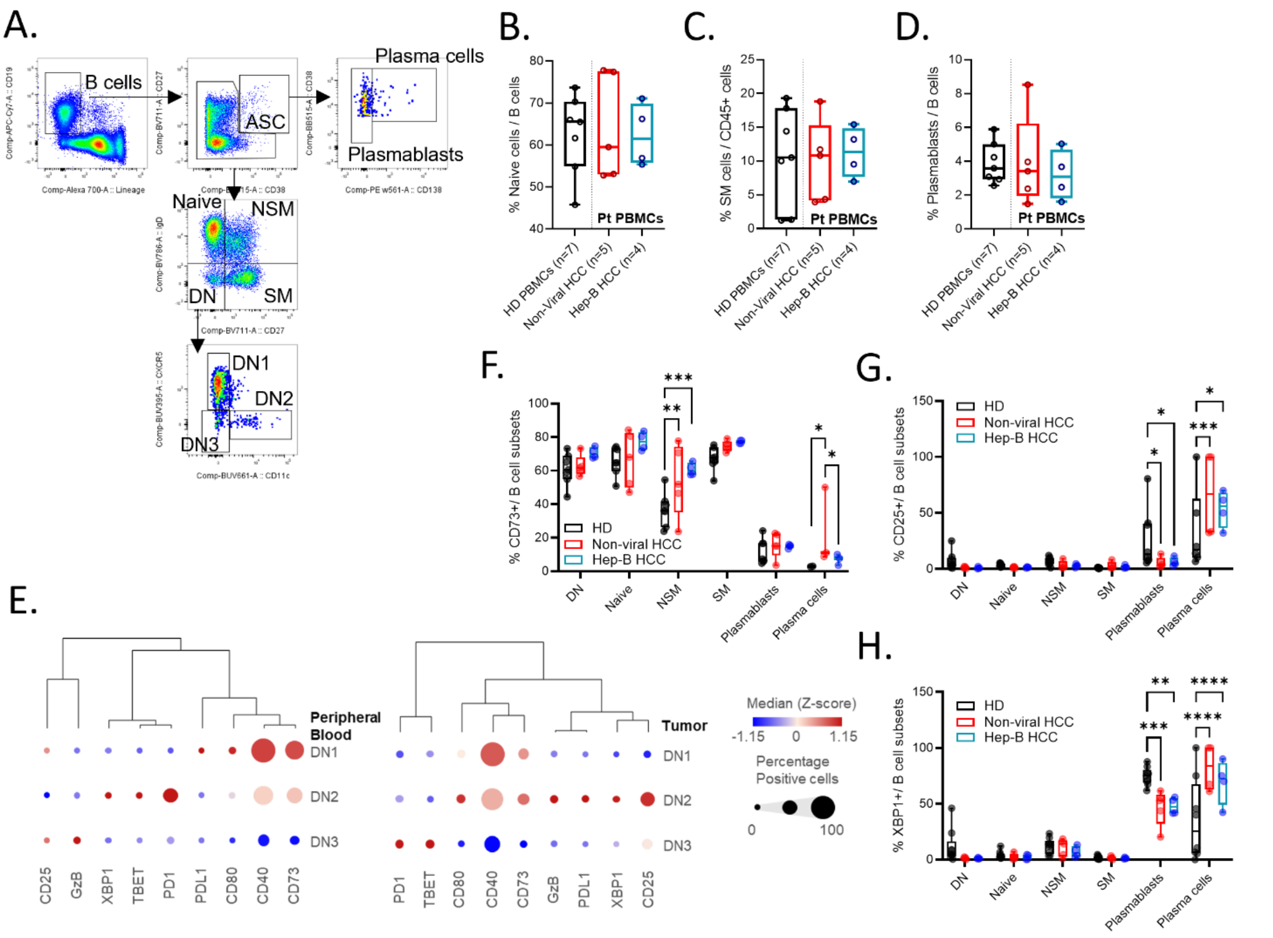

Supplementary figure S8. Differential proportions of B cell subsets within the peripheral blood of non-viral and Hepatitis B associated HCC patients.

(A) Representative flow cytometry gating strategy for plasma and B cell subsets within peripheral blood of HCC patients. Percentage of **(B)** naïve B cells, **(C)** switched memory (SM) B cells and **(D)** plasmablasts over total B cells within peripheral blood of healthy donor (HD) controls, non-viral HCC and Hep-B HCC patients. **(B to D)** Kruskal-wallis test was used for significance testing. **(E)** Bubble dot plot of functional markers expressed on the 3 DN memory B subsets in peripheral blood (Left) and Tumor (Right) of HCC patients. Differential expression of **(F)** CD73, **(G)** CD25 and **(H)** XBP1 in various B cell subsets identified in peripheral blood of healthy donors, non-viral HCC and Hep-B HCC patients. **(F to H)** One-way ANOVA was used to test for significance within each B cell subset.
